# Multi-Omics Integration-Based Prioritisation of Competing Endogenous RNA Regulation Networks in Small Cell Lung Cancer: Molecular Characteristics and Drug Candidates

**DOI:** 10.3389/fonc.2022.904865

**Published:** 2022-07-04

**Authors:** Xiao-Jun Wang, Jing Gao, Qin Yu, Min Zhang, Wei-Dong Hu

**Affiliations:** ^1^ Department of Respiratory Medicine, Gansu Provincial Hospital, Lanzhou, China; ^2^ The First School of Clinical Medicine, Lanzhou University, Lanzhou, China; ^3^ Respiratory Medicine Unit, Department of Medicine, Karolinska Institute, Stockholm, Sweden; ^4^ Department of Pulmonary Medicine, University of Helsinki and Helsinki University Hospital, Helsinki, Finland; ^5^ Department of Pathology, Gansu Provincial Hospital, Lanzhou, China

**Keywords:** small cell lung cancer (SCLC), multi-omics integration, competing endogenous RNA (ceRNA), long noncoding RNA (lncRNA), circular RNA (circRNA), microRNA (miRNA)

## Abstract

**Background:**

The competing endogenous RNA (ceRNA) network-mediated regulatory mechanisms in small cell lung cancer (SCLC) remain largely unknown. This study aimed to integrate multi-omics profiles, including the transcriptome, regulome, genome and pharmacogenome profiles, to elucidate prioritised ceRNA characteristics, pathways and drug candidates in SCLC.

**Method:**

We determined the plasma messenger RNA (mRNA), microRNA (miRNA), long noncoding RNA (lncRNA) and circular RNA (circRNA) expression levels using whole-transcriptome sequencing technology in our SCLC plasma cohort. Significantly expressed plasma mRNAs were then overlapped with the Gene Expression Omnibus (GEO) tissue mRNA data (GSE 40275, SCLC tissue cohort). Next, we applied a multistep multi-omics (transcriptome, regulome, genome and pharmacogenome) integration analysis to first construct the network and then to identify the lncRNA/circRNA-miRNA-mRNA ceRNA characteristics, genomic alterations, pathways and drug candidates in SCLC.

**Results:**

The multi-omics integration-based prioritisation of SCLC ceRNA regulatory networks consisted of downregulated mRNAs (CSF3R/GAA), lncRNAs (AC005005.4-201/DLX6-AS1-201/NEAT1-203) and circRNAs (hsa_HLA-B_1/hsa_VEGFC_8) as well as upregulated miRNAs (hsa-miR-4525/hsa-miR-6747-3p). lncRNAs (lncRNA-AC005005.4-201 and NEAT1-203) and circRNAs (circRNA-hsa_HLA-B_1 and hsa_VEGFC_8) may regulate the inhibited effects of hsa-miR-6747-3p for CSF3R expression in SCLC, while lncRNA-DLX6-AS1-201 or circRNA-hsa_HLA-B_1 may neutralise the negative regulation of hsa-miR-4525 for GAA in SCLC. CSF3R and GAA were present in the genomic alteration, and further identified as targets of FavId and Trastuzumab deruxtecan, respectively. In the SCLC-associated pathway analysis, CSF3R was involved in the autophagy pathways, while GAA was involved in the glucose metabolism pathways.

**Conclusions:**

We identified potential lncRNA/cirRNA-miRNA-mRNA ceRNA regulatory mechanisms, pathways and promising drug candidates in SCLC, providing novel potential diagnostics and therapeutic targets in SCLC.

## Introduction

Small cell lung cancer (SCLC) is a highly heterogeneous malignancy of neuroendocrine origin accounting for approximately 15% of all cases of lung cancer. SCLC is characterised by the early development of metastases, rapid recurrence and a low survival rate (1–4). The 5-year overall survival rate in SCLC barely reaches 5%, while average overall survival reaches only 2 to 4 months in untreated patients (1, 5, 6). Early diagnosis of SCLC remains quite challenging given its nonspecific symptoms and fast-growing tumours (7). Currently, chemotherapy and immunotherapy represent the most common treatment for SCLC, whereby chemotherapy alone remains the basis of standard treatment for the management of SCLC (7). While the initial response rate for first-line chemotherapy reaches approximately 60% in SCLC, patients may still quickly succumb given rapid recurrence following chemotherapy, primary or secondary drug resistance and ineffective second-line treatment options (8–10). Thus, limited effective therapies remain the primary reason for poor outcomes in SCLC (7, 8). The mechanisms behind the pathogenesis of SCLC are complex, and as yet unexplained by a single biomarker or specific mechanism (11). As such, an increased and comprehensive understanding of SCLC characteristics is crucial to guiding both diagnosis and treatment. Omics studies are emerging rapidly and offer tremendous potential to better understand the underlying disease mechanisms, as well as advancing early diagnostics and identifying potential drug targets.

Competitive endogenous RNA (ceRNA) is a novel layer of gene regulation in diseases, regulating each other at the post-transcription level by competing for shared microRNAs (miRNAs) (12). ceRNA networks link the function of protein-coding messenger RNA (mRNA) with noncoding RNAs (ncRNAs), which primarily include long noncoding RNAs (lncRNAs), circular RNAs (circRNAs) and miRNAs (12–15). The integrative assessment of the expressions of lncRNAs, circRNAs, miRNAs and mRNAs construct ceRNA networks (14–18). Several studies demonstrated that lung cancer associates with the dysregulation of the expression of ncRNAs including both lncRNAs and miRNAs, and the expression of several signalling pathways and oncogenes, while circRNAs may play a key role in lung cancer tumorigenesis, progression, invasion and metastasis (14, 18). miRNAs could control the target genes involved in cellular processes by downregulating gene expression through repressing or degrading mRNA targets (19–21). In addition, the majority of lncRNAs compete with miRNAs to prevent miRNA binding to their target mRNA, leading to the transcriptional activation of target genes (22, 23). Furthermore, after binding to several sites for a particular miRNA or RNA-binding proteins (RBPs), cirRNAs regulate alternative splicing and gene transcription through interaction (15, 23, 24). Consequently, these aberrantly expressed transcripts in the ceRNA network may represent potential therapeutic targets, diagnostic markers and prognostic markers in SCLC. In addition to transcriptomics, gene mutations play significant roles in new drug development in cancer. For instance, gene mutation profiles have facilitated the development of targeted agents in therapeutics for adenocarcinomas of the lung (25). Drug databases are developing rapidly, and the integrative analysis of omics data and drug databases provide us with excellent opportunities for drug development such as through pharmacogenomics (26). The rapidly expanding field of systems biology has proven reasonably effective at summarising knowledge related to cancer pathways, perhaps most importantly using the cancer literature to elucidate the molecular networks *via* which cancer develops. Thus, methodology which employs an integrative analysis of the literature could contribute to understanding the SCLC pathways (27).

**Figure 1:**
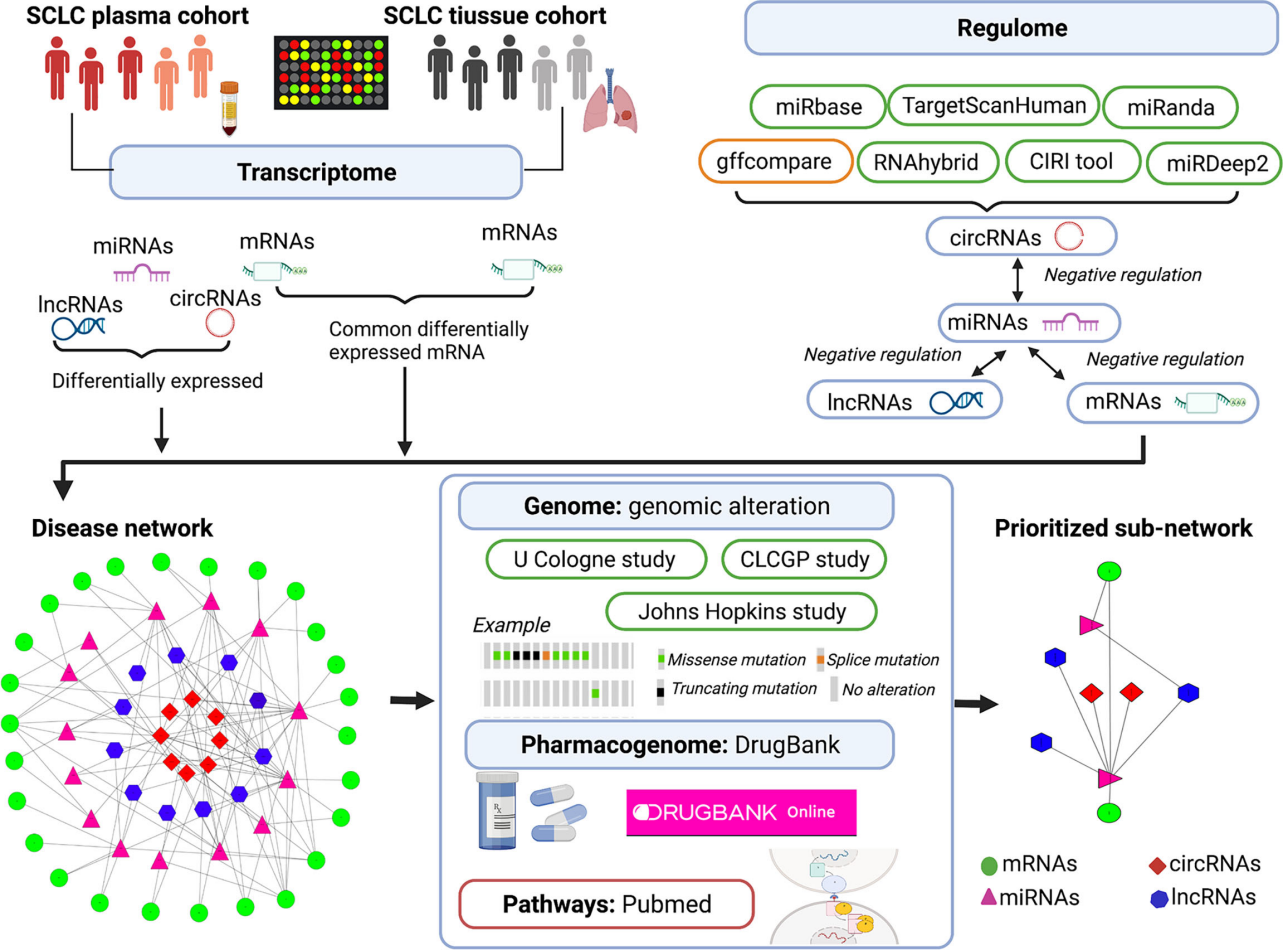
Illustration of multi-omics–based prioritisation of ceRNAs and pathways. CLCGP, Clinical Lung Cancer Genome Project; CIRI, circRNA identifier; ceRNA, competitive endogenous RNA; circRNA, circular RNAs; DE, differentially expressed; SCLC, small cell lung cancer; lncRNA, long noncoding RNA; miRNA, microRNA; mRNA, messenger RNA; cBioPortal database (https://www.cbioportal.org/datasets); DrugBank database (https://go.drugbank.com/); Genecards database (https://www.genecards.org/); PubMed (https://pubmed.ncbi.nlm.nih.gov/).

In an attempt to understand the complexity and heterogeneity of SCLC, our study aimed to identify plasma mRNAs and compare them with the expression levels found in tissue to identify SCLC-specific mRNAs (28, 29) and, further, to evaluate the lncRNA/circRNA-miRNA-mRNA ceRNA regulatory network. Next, we applied a multi-omics integration analysis (transcriptome, regulome, genome and pharmacogenome) to discuss ceRNA regulation, genomic alterations, pathways and drug candidates in SCLC (see **Figure 1**) (30–32). Understanding the characteristics of the ceRNA regulatory network can potentially shed light on the screening of SCLC biomarkers, particularly those related to genomic alterations and novel therapeutic targets.

## Materials and Methods

### In-House SCLC Plasma Cohort and SCLC Lung Tissue Cohort

In this study, we analysed two SCLC cohorts: an in-house SCLC plasma cohort (n = 12) and an SCLC lung tissue cohort (from GSE40275, n = 62) (33). The mRNA data in the SCLC tissue cohort were obtained from the lung tissue samples of SCLCs and adjacent nontumour regions. Our in-house SCLC plasma cohort includes eight SCLC patients and four healthy controls, collected between August and November 2020 at Gansu Provincial Hospital, China. The inclusion criteria of patients in our SCLC plasma cohort consisted of a histologically or cytologically confirmed initial SCLC without previous chemotherapy, radiotherapy, molecular-targeted therapy, immunotherapy or surgery. We excluded patients from our SCLC plasma cohort based on the following: (1) presence of other combined cancers; (2) pregnant or lactating patient; and (3) presentation with cardiopulmonary insufficiency, serious cardiovascular disease, a serious infection or severe malnutrition (34, 35). The mRNA data in the SCLC tissue cohort were obtained from the lung tissue samples of SCLCs and adjacent nontumour regions. In addition, the tissue mRNA expression levels were evaluated in the Gene Expression Omnibus database (GEO, https://www.ncbi.nlm.nih.gov/gds/) using the term “small cell lung cancer” with “homo sapiens”, “series” and “expression profiling by array”. The 19 SCLC lung tissue datasets were obtained, and no suitable plasma SCLC dataset could be extracted. Finally, we selected the GSE40275 tissue dataset of SCLC for further analysis, since this dataset was obtained from a single-sequencing platform, thereby avoiding a potential bias from inconsistencies in probes stemming from different sequencing platforms. This cohort study received ethical approval from the Ethics Committee of the Gansu Provincial Hospital, China (27 July 2020, No. 2020-183). Informed consent was obtained from all participants in the whole-transcriptome sequencing experiment, and the research adhered to the principles of the Declaration of Helsinki.

### Whole-Transcriptome Sequencing Analysis in the Plasma SCLC Cohort

We determined the plasma messenger RNA (mRNA), microRNA (miRNA), long noncoding RNA (lncRNA) and circular RNA (circRNA) expression levels using the whole-transcriptome sequencing technology in our SCLC plasma cohort. The extraction of total RNA from the plasma samples relied on the miRNeasy Mini Kit (Qiagen, Hilden, Germany) following the manufacturer’s protocol. The details appear in **Supplemental File 1**. A total of 1.5-μg RNA per sample was used as the input material for the lncRNA sequencing analysis, and a total of 2.5-ng RNA was used as the input material for the miRNA sequencing analysis. The details of the lncRNA and miRNA sequencing appear in **Supplemental File 1**. The steps to generating the mRNA, lncRNA, circRNA and miRNA profiles appear in **Supplemental File 2**. In addition, our SCLC plasma data were uploaded to a public platform [uploaded to the Sequence Read Archive (SRA) database (BioProject PRJNA 759049 (miRNA data) and BioProject PRJNA 762578 (mRNA, lncRNA and circRNA data)].

### Identification of Differentially Expressed mRNA, miRNA, circRNA and lncRNA in SCLC

The significant differentially expressed mRNAs (DEmRNAs) in the SCLC tissue cohort were identified by comparing SCLC lung tissue and adjacent nontumour tissue from SCLC using the GEO2R tools from the R package “limma” in GSE40275 [|fold change (FC)| > 1.5, *p* < 0.05, and false discovery rate (FDR) < 0.2)]. DEmRNAs in the SCLC plasma cohort were identified by comparing SCLC and healthy samples using the likelihood ratio test (LRT) in the R package “DESeq” (|FC| > 1.5, *p* < 0.05). Then, the commonly expressed DEmRNAs (Co-DEmRNAs, SCLC-specific mRNAs) were defined as the overlapping DEmRNAs between the SCLC plasma cohort and the SCLC lung tissue cohort (|FC| > 1.5, *p* < 0.05). The significant DEmiRNAs, DEcircRNAs and DElncRNAs in the SCLC plasma cohort were identified by comparing SCLC and healthy plasma samples using LRT in the R package “DESeq” (|FC| > 1.5, *p* < 0.05, and FDR < 0.2). FDR was computed using the methodology described by Benjamini and Hochberg (36). The volcano plots were created using the R package “ggplot2”. Finally, the Co-DEmRNAs, DEmiRNAs, DEcircRNAs and DElncRNAs were subsequently used in the ceRNA network construction.

### Construction of the lncRNA/cirRNA-miRNA-mRNA ceRNA-Mediated Regulatory Network

The previous step identifying the DEmiRNAs, DElncRNAs, DEcircRNAs and Co-DEmRNAs in SCLC was used to construct the lncRNA/circRNA-miRNA-mRNA ceRNA regulatory network. The regulome analysis was based on the targeted mRNA–miRNA, lncRNA–miRNA and circRNA–miRNA prediction using online analytical software tools. The targeted mRNAs of the miRNAs were predicted using two online analytical software tools: miRanda (version 3.3.a) (37) and TargetScanHuman database (version 5.0) (38). The targeted lncRNAs of the miRNAs were predicted using the online analytical software tools from the miRbase database (version 22.0) (37). The targeted circRNAs of the miRNAs were predicted using three online analytical software tools: RNAhybrid database (version 2.1.1) (39), miRanda (version 3.3.a) (40) and TargetScanHuman database (version 5.0) (38). The negative regulation of mRNA–miRNA, lncRNA–miRNA and circRNA–miRNA was selected in the further ceRNA network construction. Next, the lncRNAs, circRNAs and miRNAs were identified as known or novel using several analytical software tools: the gffcompare program (41), the circRNA identifier (CIRI) tool (42), the miRbase database (version 22.0) (37) and the miRDeep2 tools (43). Based on these results, we constructed the lncRNA/circRNA-miRNA-mRNA ceRNA regulatory network using the Cytoscape software (version 3.7.0) (44). Next, the differentially expressed lncRNA, circRNAs, miRNAs and mRNAs in the SCLC ceRNA network were analysed using the gene ontology (GO) analysis and the Kyoto Encyclopaedia of Genes and Genomes (KEGG) pathway analysis. For the GO analysis, the differentially expressed lncRNA, circRNAs, miRNAs and mRNAs were classified into three categories: biological process (BP), cellular component (CC) and molecular function (MF). The KEGG pathway analysis was performed to analyse the potential pathways enriched by the differentially expressed lncRNA, circRNAs, miRNAs and mRNAs. The enrichment analysis was evaluated using the R package ClusterProfiler (45), for which we considered an adjusted *p* < 0.05 as statistically significant (46).

### Evaluation of Genomic Alterations, Drug Candidates/Repurposing and Pathway Analysis in SCLC ceRNA Networks

The genomic alterations of mRNAs in the SCLC ceRNA network were determined through three datasets (47–49) from the cBioPortal database (https://www.cbioportal.org/datasets), including the Clinical Lung Cancer Genome Project (CLCGP) study (47), the Johns Hopkins study (48) and the University of Cologne study (U Cologne study) (49). The pharmacogenomics data were downloaded from the DrugBank database (release 5.0) (https://go.drugbank.com/), including the rich drugs data and the drug–target genes data (50). The results obtained from the pharmacogenomics DrugBank database were further mined through the “Targets” tool using manual searches. The pathways of the mRNAs were first evaluated and annotated using the Genecards database (https://www.genecards.org/) (51), then the SCLC-associated pathways were further filtered through a literature search from PubMed (https://pubmed.ncbi.nlm.nih.gov/) using the terms “small cell lung cancer [Title/Abstract] OR SCLC [Title/Abstract] OR small cell lung cancer [MeSH Terms]” and “pathways [Title/Abstract]”.

## Results

### Identification of Differentially Expressed mRNA, miRNA, circRNA and lncRNA in SCLC

**Figure 2:**
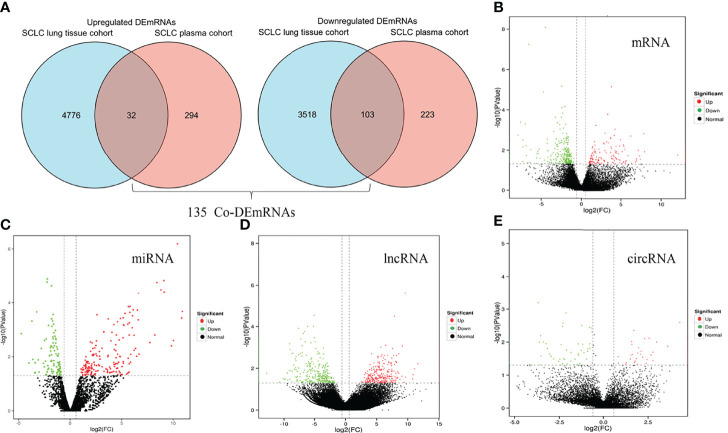
Identification of differentially expressed mRNAs, miRNAs, lncRNAs and circRNAs in SCLC. **(A)** Common differentially expressed mRNAs (Co-DEmRNAs) in the in-house SCLC plasma cohort and the SCLC lung tissue cohort (GSE40275). **(B)** Up- and downregulated mRNAs in our cohort. **(C)** Up- and downregulated miRNAs in our cohort. **(D)** Up- and downregulated lncRNAs in our cohort. **(E)** Up- and downregulated circRNAs in our cohort. Red indicates upregulated and green indicates downregulated; circRNA, circular RNAs; lncRNA, long noncoding RNA; miRNA, microRNA; mRNA, messenger RNA; SCLC, small cell lung cancer.

We identified eight SCLC patients (62.5% male, median age of 62 years, 100% Asian and 50.0% advanced stage) and four healthy controls (75.0% male, median age of 66 years) in our SCLC plasma cohort, and 19 SCLC patients (84.2% male, median age of 66 years and 100% European) in the SCLC tissue cohort (GSE40275) (**Table 1**). Through our in-house whole-transcriptome sequencing data comparing SCLC plasma samples and healthy plasma samples, we harvested a total of 652 DEmRNAs (326 upregulated and 326 downregulated), 281 DEmiRNAs (178 upregulated and 103 downregulated), 286 DEcircRNAs (166 upregulated and 120 downregulated) and 1753 DElncRNAs (1036 upregulated and 717 downregulated) for subsequent analysis. Overall, 8429 DEmRNAs (4808 upregulated and 3621 downregulated) were identified in the SCLC tissue cohort, ultimately resulting in 135 DEmRNAs (32 upregulated and 103 downregulated) expressed in two cohorts as common DEmRNAs (Co-DEmRNAs), and also identified as SCLC-specific mRNAs (**Figure 2**).

**Table 1:** Patient characteristics for the in-house SCLC plasma cohort (n = 12) and SCLC lung tissue cohort (from GSE40275, n = 62).

Patient characteristics		SCLC lung tissue cohort (GSE40275)		In-house SCLC plasma cohort	
		normal	SCLC patients	normal	SCLC patients
Age (median, in years)		66	70	66	61.5
Sex (males, %)		19 (44.2%)	16 (84.2%)	3 (75.0%)	5 (62.5%)
Country		Austria	Austria	China	China
Ethnicity		Austrian	Austrian	Asian	Asian
AJCC stage					
	Stage I	–	9 (47.4%)	–	0
	Stage II	–	4 (20.1%)	–	1 (12.5%)
	Stage III	–	6 (31.6%)	–	3 (37.5%)
	Stage IV	–	0	–	4 (50%)
VALSG stage					
	Extended stage	–	0	–	4 (50%)
	Limited stage	–	16 (100%)	–	4 (50%)
Outcome					
	Dead	–	NA	–	8 (100%)
	Living	–	NA	–	0

SCLC, small cell lung cancer; AJCC, American Joint Committee on Cancer; VALSG, Veterans Administration Lung Study Group. NA, not available.

### Construction of the lncRNA/circRNA-miRNA-mRNA ceRNA Network

**Figure 3:**
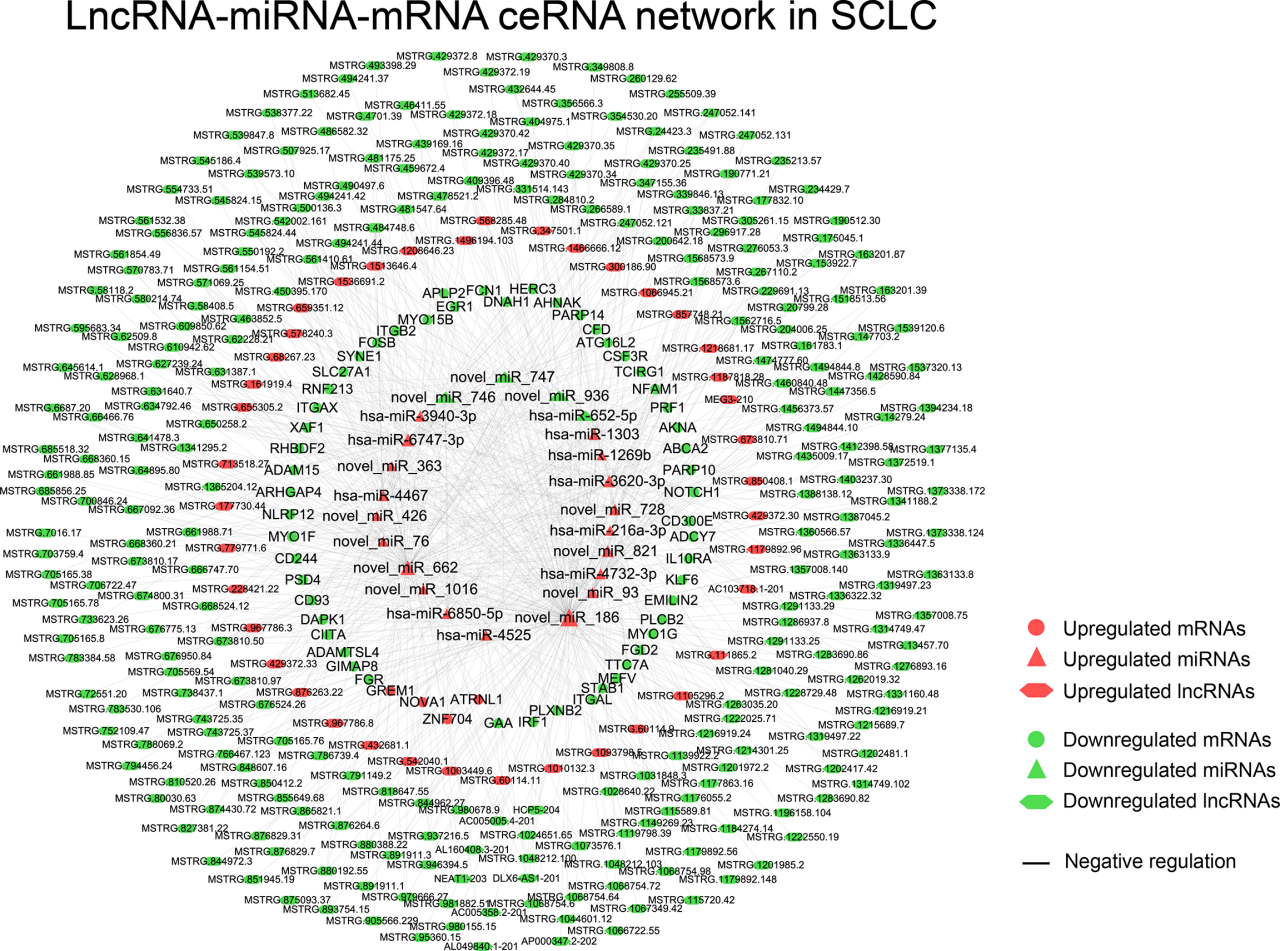
The lncRNA-miRNA-mRNA ceRNAs network in SCLC. lncRNA, long noncoding RNA; miRNA, microRNA; mRNA, messenger RNA; SCLC, small cell lung cancer.

**Figure 4:**
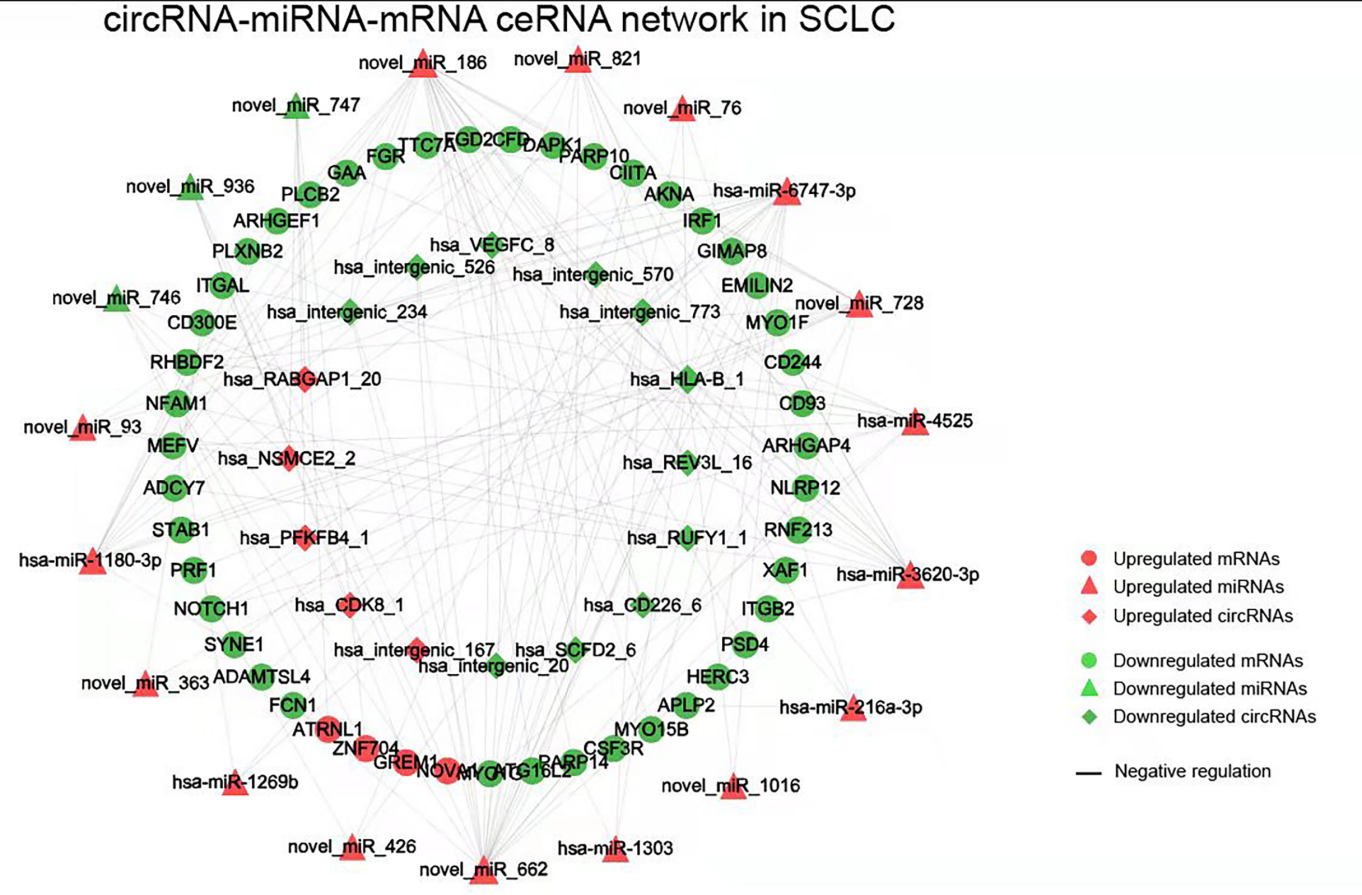
The circRNA-miRNA-mRNA ceRNAs network in SCLC. circRNA, circular RNA; miRNA, microRNA; mRNA, messenger RNA; SCLC, small cell lung cancer.

The obtained 281 DEmiRNAs, 1753 DElncRNAs, 286 DEcircRNAs and 135 Co-DEmRNAs in SCLC were initially involved in the ceRNA regulatory network construction. Integrating the selection rules described in the methods section, the SCLC lncRNA/circRNA-miRNA-mRNA ceRNA regulatory network was constructed, which included 58 mRNAs (4 upregulated and 54 downregulated), 301 lncRNAs (40 upregulated and 261 downregulated), 16 circRNAs (5 upregulated and 11 downregulated) and 24 miRNAs (20 upregulated and 4 downregulated) (**Figures 3** and **4**; **Supplemental Tables 1** and **2**). The lncRNA-miRNA-mRNA ceRNA regulatory network consisted of 381 nodes (301 lncRNAs, 23 miRNAs and 57 mRNAs) with 707 edges (**Figure 3**). In the lncRNA-miRNA-mRNA ceRNA network, the expression levels of 53 mRNAs and 261 lncRNAs decreased in SCLC and the expression levels of 19 miRNAs increased in SCLC, while the expression levels of 4 mRNAs and 40 lncRNAs increased in SCLC and the expression levels of 4 miRNAs decreased in SCLC (**Supplemental Table 1**). The circRNA-miRNA-mRNA ceRNA network consisted of 82 nodes (16 cirRNAs, 19 miRNAs and 47 mRNAs) with 165 edges (**Figure 4**). In the circRNA-miRNA-mRNA ceRNA network, the expression levels of 43 mRNAs and 11 circRNAs decreased in SCLC and the expression levels of 16 miRNAs increased in SCLC, while the expression levels of four mRNAs and five cirRNAs increased in SCLC and the expression levels of three miRNAs decreased in SCLC (**Supplemental Table 2**).

### Functional Enrichment Analysis of mRNA, miRNA, circRNA and lncRNA in the ceRNA Network in SCLC

The differentially expressed levels of 58 mRNAs in the ceRNA network appear in **Table 2**. In the SCLC plasma cohort, the top three downregulated genes in the fold change (FC) were early growth response 1 (EGR1), complement factor D (CFD) and FosB proto-oncogene AP-1 transcription factor subunit (FOSB), while the top three upregulated genes in FC were zinc finger protein 704 (ZNF704), NOVA alternative splicing regulator 1 (NOVA1) and attractin like 1 (ATRNL1) (**Table 2**). **Table 3** summarises 23 results from 58 mRNAs in the ceRNA network included in the GO analysis. This GO analysis indicated that the DEmRNAs were associated with numerous important biological processes and cellular components. The present study indicated that the biological processes of DEmRNAs primarily included processes such as neutrophil degranulation, neutrophil activation involved in the immune response, neutrophil activation, neutrophil-mediated immunity and an integrin-mediated signalling pathway among others. These biological functions associate with the protumour/prometastatic roles of inflammatory cells in cancer development and metastasis (**Table 3**) (52, 53). In terms of the cellular components, they mainly included the protein complex involved in cell adhesion and the integrin complex (**Table 3**), functions associated with tumorigenesis (54, 55). In addition, no results were obtained from the molecular function of the GO analysis and the KEGG pathways analysis, given that adjusted *p* > 0.05 in these functional analyses. In addition, we also reported the differentially expressed levels of lncRNAs, circRNAs and miRNAs in the ceRNA network (**Supplemental Tables 3**-**5**). The functional GO analyses primarily revealed cell survival and proliferation in 42 functional results from 301 lncRNAs, the inflammatory and immune response function in 32 functional results from 32 circRNAs and inflammatory and immune response and cell proliferation in 66 functional results from 24 miRNAs, respectively (**Tables 4**–**6**). Among these functions, many tumour-related terms were significantly enriched, such as regulating the cell cycle, the negative regulation of cell growth, DNA recombination and the MyD88-independent toll-like receptor signalling pathway, as well as the regulation of dendritic cell differentiation. In the KEGG pathways analyses, five pathways were identified in the lncRNAs, consisting of olfactory transduction, the neuroactive ligand–receptor interaction, nicotine addiction, carbohydrate digestion and absorption, and the protein digestion and absorption pathway (**Table 4**). The 60 pathways found in the miRNAs and mainly tumour-related pathways were significantly enriched, including the cAMP signalling pathway, focal adhesion, the MAPK signalling pathway, the Hippo signalling pathway and the ECM–receptor interaction (**Table 7**).

**Table 2:** Differentially expressed levels and genomic alterations of mRNAs in the ceRNA regulatory network in SCLC.

Gene symbol	Gene full name	Differentially expressed levels					Genomic alterations		
		In-house SCLC plasma cohort		SCLC lung tissue cohort (GSE40275)		Regulated	CLCGP, *Nat Genet* 2012	Johns Hopkins, *Nat Genet* 2012	U Cologne,*Nature* 2015
		log2FC	*p* value	log2FC	*p* value				
Genomic alterations (n = 50)									
EGR1	Early Growth Response 1	-3.232	2.30E-04	-2.611	3.42E-16	down	3.0%	0	0
CFD	Complement Factor D	-2.898	2.11E-02	-2.472	2.01E-25	down	0	0	0.8%
ABCA2	ATP Binding Cassette Subfamily A Member 2	-2.814	3.54E-03	-1.923	3.00E-04	down	3.0%	1.3%	2.5%
PRF1	Perforin 1	-2.699	5.32E-04	-2.038	5.60E-20	down	3.0%	0	1.7%
STAB1	Stabilin 1	-2.484	2.34E-04	-1.151	2.39E-14	down	3.0%	0	4.0%
AHNAK	AHNAK Nucleoprotein	-2.443	6.74E-06	-2.761	8.93E-29	down	7.0%	4.0%	6.0%
CD300E	CD300e Molecule	-2.428	7.77E-05	-1.009	3.72E-15	down	0	0	0.8%
CD244	CD244 Molecule	-2.332	2.57E-02	-0.751	3.88E-15	down	3.0%	0	0.8%
SLC27A1	Solute Carrier Family 27 Member 1	-2.331	3.90E-02	-0.76	6.68E-14	down	7.0%	1.3%	1.7%
PARP10	Poly (ADP-Ribose) Polymerase Family Member 10	-2.12	2.62E-02	-0.601	4.27E-10	down	3.0%	0	0.8%
MEFV	MEFV Innate Immuity Regulator, Pyrin	-2.051	9.52E-03	-1.031	2.62E-19	down	0	1.3%	1.7%
RHBDF2	Rhomboid 5 Homolog 2	-2.028	3.29E-02	-0.981	5.62E-14	down	3.0%	0	0
DNAH1	Dynein Axonemal Heavy Chain 1	-2.02	1.14E-02	-0.653	2.03E-18	down	0	0	7.0%
TCIRG1	T Cell Immune Regulator 1, ATPase H+ Transporting V0 Subunit A3	-1.998	9.10E-03	-1.421	4.46E-17	down	0	0	1.7%
NFAM1	NFAT Activating Protein With ITAM Motif 1	-1.976	4.41E-02	-0.708	2.61E-13	down	0	1.3%	0.8%
GIMAP8	GTPase, IMAP Family Member 8	-1.902	1.10E-02	-2.078	2.10E-31	down	10.0%	0	3.0%
PLXNB2	Plexin B2	-1.896	3.39E-03	-1.094	1.38E-09	down	7.0%	1.3%	3.0%
FGD2	FYVE, RhoGEF And PH Domain Containing 2	-1.885	3.07E-03	-1.384	7.07E-19	down	0	0	0.8%
NLRP12	NLR Family Pyrin Domain Containing 12	-1.862	2.96E-02	-0.852	4.45E-16	down	7.0%	1.3%	4.0%
NOTCH1	Notch Receptor 1	-1.848	2.58E-02	-1.497	3.76E-22	down	10.0%	1.3%	13.0%
FCN1	Ficolin 1	-1.844	7.66E-03	-1.675	3.00E-23	down	0	0	2.5%
CSF3R	Colony-stimulating factor 3 receptor	-1.801	2.63E-03	-2.469	2.01E-29	down	7.0%	1.3%	2.5%
GAA	Acid alpha-glucosidase	-1.789	3.85E-02	-1.108	5.29E-13	down	3.0%	1.3%	2.5%
ITGB2	Integrin Subunit Beta 2	-1.756	9.89E-03	-1.813	1.26E-11	down	3.0%	0	2.5%
EMILIN2	Elastin Microfibril Interfacer 2	-1.748	8.86E-03	-1.372	1.79E-18	down	0	2.5%	2.5%
ARHGAP4	Rho GTPase Activating Protein 4	-1.741	1.37E-02	-0.624	3.80E-07	down	3.0%	1.3%	4.0%
CD93	CD93 Molecule	-1.722	2.15E-02	-2.668	4.54E-34	down	3.0%	0	1.7%
DAPK1	Death Associated Protein Kinase 1	-1.707	1.97E-02	-1.123	5.50E-05	down	3.0%	2.5%	4.0%
TTC7A	Tetratricopeptide Repeat Domain 7A	-1.651	2.83E-02	-1.265	3.85E-20	down	0	1.3%	2.5%
PSD4	Pleckstrin And Sec7 Domain Containing 4	-1.632	1.74E-02	-0.802	3.80E-11	down	3.0%	1.3%	3.0%
CIITA	Class II Major Histocompatibility Complex Transactivator	-1.624	2.17E-03	-1.777	3.50E-17	down	0	1.3%	0
SYNE1	Spectrin Repeat Containing Nuclear Envelope Protein 1	-1.606	3.16E-03	-1.689	1.78E-19	down	28.0%	11.0%	23.0%
ITGAX	Integrin Subunit Alpha X	-1.592	1.15E-02	-2.083	9.75E-18	down	3.0%	1.3%	3.0%
ADAMTSL4	ADAMTS Like 4	-1.555	3.60E-02	-1.606	2.64E-22	down	0	0	2.5%
XAF1	XIAP Associated Factor 1	-1.552	1.88E-02	-1.445	3.44E-10	down	3.0%	1.3%	0
FGR	FGR Proto-Oncogene, Src Family Tyrosine Kinase	-1.488	2.02E-02	-2.179	5.88E-22	down	0	2.5%	0.8%
PLCB2	Phospholipase C Beta 2	-1.474	1.89E-02	-1.634	8.74E-19	down	0	1.3%	0
APLP2	Amyloid Beta Precursor Like Protein 2	-1.47	2.22E-02	-0.935	5.30E-17	down	5.0%	2.5%	0
AKNA	AT-Hook Transcription Factor	-1.467	4.69E-02	-1.126	7.79E-20	down	7.0%	2.5%	1.7%
RNF213	Ring Finger Protein 213	-1.452	1.46E-02	-0.714	8.47E-06	down	0	4.0%	2.5%
HERC3	HECT And RLD Domain Containing E3 Ubiquitin Protein Ligase 3	-1.45	4.01E-02	-0.725	1.92E-16	down	0	0	0.8%
ARHGEF1	Rho Guanine Nucleotide Exchange Factor 1	-1.443	3.72E-02	-0.724	6.28E-09	down	0	1.3%	2.5%
MYO1F	Myosin 1F	-1.394	4.04E-02	-2.014	2.90E-22	down	3.0%	1.3%	2.5%
MYO1G	Myosin 1G	-1.314	3.45E-02	-1.526	3.29E-20	down	3.0%	0	1.7%
ADCY7	Adenylate Cyclase 7	-1.314	3.64E-02	-1.626	7.20E-23	down	3.0%	0	4.0%
PARP14	Poly(ADP-Ribose) Polymerase Family Member 14	-1.233	4.09E-02	-1.178	2.66E-08	down	0	0	2.5%
ITGAL	Integrin Subunit Alpha L	-1.225	3.83E-02	-1.893	6.38E-17	down	3.0%	0	5.0%
ZNF704	Zinc Finger Protein 704	Inf	2.00E-02	1.059	8.34E-13	up	0	0	0.8%
NOVA1	NOVA Alternative Splicing Regulator 1	Inf	3.63E-02	1.039	2.61E-16	up	0	1.3%	1.7%
ATRNL1	Attractin Like 1	Inf	3.34E-02	0.878	6.12E-09	up	7.0%	4.0%	3.0%
No genomic alterations (n = 8)									
FOSB	FosB Proto-Oncogene, AP-1 Transcription Factor Subunit	-3.723	4.45E-02	-3.385	2.01E-15	down	0	0	0
ADAM15	ADAM Metallopeptidase Domain 15	-3.479	1.29E-02	-0.586	4.13E-08	down	0	0	0
KLF6	Kruppel Like Factor 6	-1.999	4.21E-03	-1.665	3.59E-18	down	0	0	0
IL10RA	Interleukin 10 Receptor Subunit Alpha	-1.921	2.54E-03	-1.772	8.82E-14	down	0	0	0
ATG16L2	Autophagy Related 16 Like 2	-1.851	0.01755	-0.665	1.89E-12	down	0	0	0
MYO15B	Myosin XVB	-1.814	4.40E-02	-0.837	1.26E-12	down	0	0	0
IRF1	Interferon Regulatory Factor 1	-1.589	8.63E-03	-1.775	4.68E-11	down	0	0	0
GREM1	Gremlin 1, DAN Family BMP Antagonist	Inf	4.60E-02	1.011	1.16E-08	up	0	0	0

SCLC, small cell lung cancer; circRNA, circular RNA; lncRNA, long noncoding RNA; miRNA, microRNA; mRNA, messenger RNA; ceRNA, competing endogenous RNA; FC, fold change; Inf, infinity; CLCGP, Clinical Lung Cancer Genome Project; U Cologne, University of Cologne study.

**Table 3:** Functional enrichment analysis of mRNAs in the ceRNA network in SCLC.

ID	Description	Ontology	Bg Ratio	*p* value	Adjusted *p*	Genes symbol*	Count
GO:0043312	neutrophil degranulation	BP	485/18670	1.843E-06	9.114E-04	CFD/FCN1/FGR/GAA/ITGAL/ITGAX/ITGB2/TCIRG1/CD93/NFAM1	10
GO:0002283	neutrophil activation involved in immune response	BP	488/18670	1.948E-06	9.114E-04	CFD/FCN1/FGR/GAA/ITGAL/ITGAX/ITGB2/TCIRG1/CD93/NFAM1	10
GO:0042119	neutrophil activation	BP	498/18670	2.336E-06	9.114E-04	CFD/FCN1/FGR/GAA/ITGAL/ITGAX/ITGB2/TCIRG1/CD93/NFAM1	10
GO:0002446	neutrophil-mediated immunity	BP	499/18670	2.378E-06	9.114E-04	CFD/FCN1/FGR/GAA/ITGAL/ITGAX/ITGB2/TCIRG1/CD93/NFAM1	10
GO:0007229	integrin-mediated signalling pathway	BP	103/18670	1.545E-05	4.738E-03	FGR/ITGAL/ITGAX/ITGB2/ADAM15	5
GO:0050663	cytokine secretion	BP	240/18670	8.892E-05	2.272E-02	FCN1/FGR/NOTCH1/TCIRG1/CD244/NLRP12	6
GO:0030198	extracellular matrix organization	BP	368/18670	1.237E-04	2.467E-02	ITGAL/ITGAX/ITGB2/NOTCH1/ADAM15/GREM1/ADAMTSL4	7
GO:0050900	leukocyte migration	BP	499/18670	1.287E-04	2.467E-02	CSF3R/ITGAL/ITGAX/ITGB2/GREM1/CD244/MYO1G/NLRP12	8
GO:0043062	extracellular structure organization	BP	422/18670	2.861E-04	4.873E-02	ITGAL/ITGAX/ITGB2/NOTCH1/ADAM15/GREM1/ADAMTSL4	7
GO:0101003	ficolin-1-rich granule membrane	CC	61/19717	8.873E-07	6.558E-05	GAA/ITGAX/ITGB2/TCIRG1/CD93	5
GO:0101002	ficolin-1-rich granule	CC	185/19717	1.017E-06	6.558E-05	CFD/FCN1/GAA/ITGAX/ITGB2/TCIRG1/CD93	7
GO:0030667	secretory granule membrane	CC	298/19717	2.154E-06	9.263E-05	APLP2/GAA/ITGAL/ITGAX/ITGB2/TCIRG1/CD93/NFAM1	8
GO:0070821	tertiary granule membrane	CC	73/19717	5.888E-05	1.899E-03	GAA/ITGAX/ITGB2/CD93	4
GO:0008305	integrin complex	CC	31/19717	9.721E-05	2.370E-03	ITGAL/ITGAX/ITGB2	3
GO:0070820	tertiary granule	CC	164/19717	1.106E-04	2.370E-03	GAA/ITGAX/ITGB2/TCIRG1/CD93	5
GO:0098636	protein complex involved in cell adhesion	CC	34/19717	1.286E-04	2.370E-03	ITGAL/ITGAX/ITGB2	3
GO:0005774	vascular membrane	CC	412/19717	1.184E-03	1.910E-02	ABCA2/GAA/TCIRG1/AHNAK/ATG16L2/NFAM1	6
GO:0031256	leading edge membrane	CC	170/19717	1.476E-03	1.988E-02	FGR/PSD4/MYO1G/FGD2	4
GO:0001726	Ruffle	CC	172/19717	1.541E-03	1.988E-02	FGR/MEFV/PSD4/FGD2	4
GO:0035579	specific granule membrane	CC	91/19717	2.324E-03	2.725E-02	ITGAL/ITGB2/CD93	3
GO:0032587	ruffle membrane	CC	94/19717	2.549E-03	2.740E-02	FGR/PSD4/FGD2	3
GO:0005765	lysosomal membrane	CC	354/19717	3.536E-03	3.297E-02	ABCA2/GAA/TCIRG1/AHNAK/NFAM1	5
GO:0098852	lytic vacuole membrane	CC	355/19717	3.578E-03	3.297E-02	ABCA2/GAA/TCIRG1/AHNAK/NFAM1	5

GO, gene ontology; BP, biological process; CC, cellular component; KEGG, Kyoto Encyclopaedia of Genes and Genomes; ceRNA, competing endogenous RNA; circRNA, circular RNAs; lncRNA, long noncoding RNA; miRNA, microRNA; mRNA, messenger RNA; SCLC, small cell lung cancer; Bg, background. *The full name of gene symbols is available in **Table 2**.

**Table 4:** Functional enrichment analysis and pathway results of lncRNAs in the ceRNA network.

ID	Description	Ontology	Bg Ratio	p value	Adjusted p
GO:0050911	Detection of chemical stimulus involved in sensory perception of smell	BP	0.0252	2.5259E-19	1.5494E-15
GO:0032199	Reverse transcription involved in RNA-mediated transposition	BP	0.0486	2.0772E-15	6.3707E-12
GO:0090305	Nucleic acid phosphodiester bond hydrolysis	BP	0.058	2.5433E-14	5.2002E-11
GO:0007186	G-protein coupled receptor signalling pathway	BP	0.0406	7.6252E-13	1.1693E-09
GO:0097252	Oligodendrocyte apoptotic process	BP	0.039	1.6472E-11	2.0208E-08
GO:0006289	Nucleotide-excision repair	BP	0.0402	4.2385E-11	4.3332E-08
GO:0090200	Positive regulation of release of cytochrome c from mitochondria	BP	0.0399	8.0612E-11	6.7949E-08
GO:0000733	DNA strand renaturation	BP	0.0395	8.8620E-11	6.7949E-08
GO:0007569	Cell aging	BP	0.0397	1.1273E-10	7.6831E-08
GO:0030308	Negative regulation of cell growth	BP	0.0447	1.8945E-10	1.1621E-07
GO:0007275	Multicellular organism development	BP	0.0664	2.2891E-08	1.2765E-05
GO:0006310	DNA recombination	BP	0.0325	3.6143E-08	1.8475E-05
GO:0006278	RNA-dependent DNA biosynthetic process	BP	0.0088	4.2840E-08	2.0214E-05
GO:0032197	Transposition, RNA-mediated	BP	0.0081	6.9777E-06	3.0572E-03
GO:0009987	Cellular process	BP	0.003	1.2068E-05	4.9352E-03
GO:0006259	DNA metabolic process	BP	0.0054	1.4735E-05	5.6492E-03
GO:0007156	Homophilic cell adhesion *via* plasma membrane adhesion molecules	BP	0.0098	7.3718E-05	2.6599E-02
GO:0016043	Cellular component organisation	BP	0.0064	7.8691E-05	2.6816E-02
GO:0044238	Primary metabolic process	BP	0.0027	1.3610E-04	4.3939E-02
GO:0048741	Skeletal muscle fibre development	BP	0.0141	1.6591E-04	4.8714E-02
GO:0003338	Metanephros morphogenesis	BP	0.001	1.7472E-04	4.8714E-02
GO:0070307	Lens fibre cell development	BP	0.001	1.7472E-04	4.8714E-02
GO:0044424	Intracellular part	CC	0.007	1.7884E-07	1.6189E-04
GO:0043229	Intracellular organelle	CC	0.0019	1.2468E-06	4.2980E-04
GO:0005886	Plasma membrane	CC	0.1378	1.4243E-06	4.2980E-04
GO:0044446	Intracellular organelle part	CC	0.0032	2.8392E-06	6.4257E-04
GO:0098588	Bounding membrane of organelle	CC	0.0086	5.0429E-05	9.1302E-03
GO:0044456	Synapse part	CC	0.0013	1.3285E-04	1.9921E-02
GO:0005739	Mitochondrion	CC	0.0821	1.6615E-04	1.9921E-02
GO:0005796	Golgi lumen	CC	0.0066	1.7604E-04	1.9921E-02
GO:0005578	Proteinaceous extracellular matrix	CC	0.0098	2.3813E-04	2.3264E-02
GO:0016021	Integral component of membrane	CC	0.2479	2.5699E-04	2.3264E-02
GO:0097546	Ciliary base	CC	0.0041	5.4612E-04	4.4944E-02
GO:0005887	Integral component of plasma membrane	CC	0.066	6.2753E-04	4.7340E-02
GO:0003964	RNA-directed DNA polymerase activity	MF	0.0534	7.1861E-20	1.0143E-16
GO:0004984	Olfactory receptor activity	MF	0.0249	1.0694E-19	1.0143E-16
GO:0004930	G-protein coupled receptor activity	MF	0.0316	4.2156E-17	2.6656E-14
GO:0009036	Type II site-specific deoxyribonuclease activity	MF	0.0479	1.2775E-16	6.0586E-14
GO:0005507	Copper ion binding	MF	0.0408	1.0171E-10	3.8588E-08
GO:0005488	Binding	MF	0.0105	1.9980E-10	6.3171E-08
GO:0043167	Ion binding	MF	0.0116	3.3737E-07	9.1428E-05
GO:0005549	Odorant binding	MF	0.0056	1.5499E-06	3.6752E-04
hsa04740	Olfactory transduction	KEGG	0.0598	8.0910E-40	2.2399E-37
hsa04080	Neuroactive ligand-receptor interaction	KEGG	0.0385	5.7920E-08	8.0173E-06
hsa05033	Nicotine addiction	KEGG	0.0054	2.1224E-04	1.9585E-02
hsa04973	Carbohydrate digestion and absorption	KEGG	0.0076	5.8461E-04	3.3090E-02
hsa04974	Protein digestion and absorption	KEGG	0.012	5.9763E-04	3.3090E-02

GO, gene ontology; BP, biological process; CC, cellular component; KEGG, Kyoto Encyclopaedia of Genes and Genomes; ceRNA, competing endogenous RNA; circRNA, circular RNAs; lncRNA, long noncoding RNA; miRNA, microRNA; mRNA, messenger RNA; Bg, background.

**Table 5:** Functional enrichment analysis of circRNAs in the ceRNA network.

ID	Description	Ontology	Bg Ratio	*p* value	Adjusted *p*
GO:0032655	regulation of interleukin-12 production	BP	0.0001	2.963E-04	3.119E-04
GO:0032675	regulation of interleukin-6 production	BP	0.0001	2.963E-04	3.119E-04
GO:2001198	regulation of dendritic cell differentiation	BP	0.0001	2.963E-04	3.119E-04
GO:0002667	regulation of T cell anergy	BP	0.0002	8.888E-04	7.017E-04
GO:0002486	antigen processing and presentation of endogenous peptide antigen *via* MHC class I *via* ER pathway, TAP-independent	BP	0.0004	1.481E-03	8.311E-04
GO:0015031	protein transport	BP	0.0175	1.784E-03	8.311E-04
GO:0001916	positive regulation of T cell–mediated cytotoxicity	BP	0.0005	2.073E-03	8.311E-04
GO:0016045	detection of bacterium	BP	0.0006	2.369E-03	8.311E-04
GO:0042270	protection from natural killer cell–mediated cytotoxicity	BP	0.0006	2.369E-03	8.311E-04
GO:0002480	antigen processing and presentation of exogenous peptide antigen *via* MHC class I, TAP-independent	BP	0.0007	2.664E-03	8.414E-04
GO:0030100	regulation of endocytosis	BP	0.0010	3.847E-03	1.104E-03
GO:0006904	vesicle docking involved in exocytosis	BP	0.0011	4.438E-03	1.168E-03
GO:0060337	type I interferon signalling pathway	BP	0.0022	8.861E-03	2.152E-03
GO:0002479	antigen processing and presentation of exogenous peptide antigen *via* MHC class I, TAP-independent	BP	0.0030	1.180E-02	2.546E-03
GO:0060333	interferon gamma–mediated signalling pathway	BP	0.0030	1.210E-02	2.546E-03
GO:0051726	regulation of cell cycle	BP	0.0074	2.931E-02	5.784E-03
GO:0006367	transcription initiation from RNA polymerase II promoter	BP	0.0108	4.257E-02	7.908E-03
GO:0006468	protein phosphorylation	BP	0.0122	4.801E-02	8.423E-03
GO:0031901	early endosome membrane	CC	0.0062	1.137E-04	5.983E-04
GO:0042612	MHC class I protein complex	CC	0.0010	2.892E-03	6.082E-03
GO:0016592	mediator complex	CC	0.0017	4.954E-03	6.082E-03
GO:0071556	integral component of the lumenal side of endoplasmic reticulum membrane	CC	0.0017	4.954E-03	6.082E-03
GO:0012507	ER to Golgi transport vesicle membrane	CC	0.0019	5.778E-03	6.082E-03
GO:0030670	phagocytic vesicle membrane	CC	0.0028	8.454E-03	7.415E-03
GO:0046977	TAP binding	MF	0.0004	1.093E-03	3.107E-03
GO:0008353	RNA polymerase II carboxy-terminal domain kinase activity	MF	0.0007	1.967E-03	3.107E-03
GO:0004693	cyclin-dependent protein serine/threonine kinase activity	MF	0.0020	6.113E-03	5.857E-03
GO:0042605	peptide antigen binding	MF	0.0025	7.419E-03	5.857E-03
GO:0051087	chaperone binding	MF	0.0039	1.155E-02	6.307E-03
GO:0008565	protein transporter activity	MF	0.0040	1.198E-02	6.307E-03
GO:0008289	lipid binding	MF	0.0061	1.826E-02	8.239E-03
GO:0005102	receptor binding	MF	0.0102	3.031E-02	1.197E-02

GO, gene ontology; BP, biological process; CC, cellular component; ceRNA, competing endogenous RNA; circRNA, circular RNAs; Bg, background.

**Table 6:** Functional enrichment analysis of miRNAs in the ceRNA network.

ID	Description	Ontology	Bg Ratio	*p* value	Adjusted *p*
GO:0006355	regulation of transcription, DNA-templated	BP	0.0921	8.5782E-11	5.7934E-07
GO:0000122	negative regulation of transcription from RNA polymerase II promoter	BP	0.0565	1.5250E-08	5.1498E-05
GO:0045944	positive regulation of transcription from RNA polymerase II promoter	BP	0.0664	2.6517E-08	5.9696E-05
GO:0060348	bone development	BP	0.0174	6.6166E-08	1.0284E-04
GO:0017144	drug metabolic process	BP	0.0187	8.6063E-08	1.0284E-04
GO:0017187	peptidyl-glutamic acid carboxylation	BP	0.0179	9.1359E-08	1.0284E-04
GO:0042373	vitamin K metabolic process	BP	0.0177	2.6291E-07	2.5366E-04
GO:0007250	activation of NF-kappa-inducing kinase activity	BP	0.0124	3.1140E-07	2.5645E-04
GO:0007156	hemophilic cell adhesion *via* plasma membrane adhesion molecules	BP	0.0098	3.4174E-07	2.5645E-04
GO:0032743	positive regulation of interleukin 2 production	BP	0.0125	6.9433E-07	4.6893E-04
GO:2000679	positive regulation of transcription regulatory region DNA binding	BP	0.017	1.4950E-06	9.1787E-04
GO:0031293	membrane protein intracellular domain proteolysis	BP	0.0124	2.1866E-06	1.2306E-03
GO:0002756	MyD88-independent toll-like receptor signalling pathway	BP	0.0116	3.2308E-06	1.6785E-03
GO:0000187	activation of MAPK activity	BP	0.0213	5.9088E-06	2.8504E-03
GO:0002726	positive regulation of T cell cytokine production	BP	0.0124	9.3027E-06	4.1885E-03
GO:0070555	response to interleukin 1	BP	0.0098	1.0189E-05	4.2601E-03
GO:0051865	protein auto-ubiquitination	BP	0.0143	1.0723E-05	4.2601E-03
GO:0045672	positive regulation of osteoclast differentiation	BP	0.0125	1.3074E-05	4.7509E-03
GO:0001932	regulation of protein phosphorylation	BP	0.003	1.4009E-05	4.7509E-03
GO:0070534	protein K63-linked ubiquitination	BP	0.0182	1.4069E-05	4.7509E-03
GO:0031398	positive regulation of protein ubiquitination	BP	0.0121	2.6984E-05	8.2836E-03
GO:0034162	toll-like receptor 9 signalling pathway	BP	0.0121	2.6984E-05	8.2836E-03
GO:0070423	nucleotide-binding oligomerisation domain containing signalling pathway	BP	0.0175	2.8472E-05	8.3605E-03
GO:0043507	positive regulation of JUN kinase activity	BP	0.0139	4.0463E-05	1.1386E-02
GO:0030574	collagen catabolic process	BP	0.0023	4.2766E-05	1.1553E-02
GO:0071222	cellular response to lipopolysaccharide	BP	0.0118	5.4294E-05	1.4070E-02
GO:0002755	MyD88-dependent toll-like receptor signalling pathway	BP	0.0181	5.6249E-05	1.4070E-02
GO:0046513	ceramide biosynthetic process	BP	0.0096	6.7342E-05	1.6134E-02
GO:0035019	somatic stem cell population maintenance	BP	0.0075	6.9279E-05	1.6134E-02
GO:0001707	mesoderm formation	BP	0.0013	8.3053E-05	1.8697E-02
GO:0007596	blood coagulation	BP	0.0236	9.1112E-05	1.9850E-02
GO:0050870	positive regulation of T cell activation	BP	0.0067	1.5077E-04	3.1820E-02
GO:0007155	cell adhesion	BP	0.0111	1.5634E-04	3.1997E-02
GO:0015886	heme transport	BP	0.0035	1.6785E-04	3.2879E-02
GO:0043065	positive regulation of apoptotic process	BP	0.028	1.7039E-04	3.2879E-02
GO:0045059	positive thymic T cell selection	BP	0.0023	1.9247E-04	3.6108E-02
GO:0035023	regulation of Rho protein signal transduction	BP	0.0039	2.1384E-04	3.9032E-02
GO:0051092	positive regulation of NF-kappa B transcription factor activity	BP	0.026	2.2701E-04	4.0346E-02
GO:0031410	cytoplasmic vesicle	CC	0.014	8.1251E-07	6.2029E-04
GO:0005789	endoplasmic reticulum membrane	CC	0.0602	1.2645E-06	6.2029E-04
GO:0010008	endosome membrane	CC	0.0227	1.8113E-06	6.2029E-04
GO:0005829	cytosol	CC	0.1935	7.5017E-06	1.9267E-03
GO:0034704	calcium channel complex	CC	0.0008	5.8805E-05	1.2083E-02
GO:0005811	lipid droplet	CC	0.012	7.5151E-05	1.2868E-02
GO:0035631	CD40 receptor complex	CC	0.0098	1.0108E-04	1.3848E-02
GO:0009898	cytoplasmic side of plasma membrane	CC	0.0116	1.0783E-04	1.3848E-02
GO:0005667	transcription factor complex	CC	0.0095	3.8115E-04	4.3509E-02
GO:0003700	transcription factor activity, sequence-specific DNA binding	MF	0.0684	1.2784E-14	2.7816E-11
GO:0000977	RNA polymerase II regulatory region sequence-specific DNA binding	MF	0.0261	6.2083E-13	6.7539E-10
GO:0046872	metal ion binding	MF	0.1355	1.8538E-08	1.3445E-05
GO:0031996	thioesterase binding	MF	0.0128	1.6534E-07	7.5276E-05
GO:0031624	ubiquitin conjugating enzyme binding	MF	0.0136	1.7299E-07	7.5276E-05
GO:0042826	histone deacetylase binding	MF	0.0191	3.5880E-07	1.3011E-04
GO:0047057	vitamin-K-epoxide reductase (warfarin-sensitive) activity	MF	0.0174	4.3664E-07	1.3572E-04
GO:0043422	protein kinase B binding	MF	0.0122	5.3369E-07	1.4515E-04
GO:0031435	mitogen-activated protein kinase binding	MF	0.0125	2.4614E-06	5.9505E-04
GO:0005164	tumour necrosis factor receptor binding	MF	0.0133	4.8342E-06	1.0518E-03
GO:0050291	sphingosine N-acyltransferase activity	MF	0.0083	2.3602E-05	4.6685E-03
GO:0003682	chromatin binding	MF	0.0168	3.5302E-05	6.4008E-03
GO:0001077	transcriptional activator activity, RNA polymerase II core promoter proximal region sequence-specific binding	MF	0.0219	4.3610E-05	7.2989E-03
GO:0005096	GTPase activator activity	MF	0.0123	1.0248E-04	1.5927E-02
GO:0031625	ubiquitin protein ligase binding	MF	0.0317	1.2898E-04	1.8708E-02
GO:0001078	transcriptional repressor activity, RNA polymerase II core promoter proximal region sequence-specific binding	MF	0.009	1.5448E-04	2.1007E-02
GO:0000978	RNA polymerase II core promoter proximal region sequence-specific DNA binding	MF	0.0242	1.9703E-04	2.5217E-02
GO:0008270	zinc ion binding	MF	0.0636	2.6201E-04	3.1672E-02
GO:0001047	core promoter binding	MF	0.0109	4.3480E-04	4.9792E-02

GO, gene ontology; BP, biological process; CC, cellular component; ceRNA, competing endogenous RNA; miRNA, microRNA; Bg, background.

**Table 7:** Pathway results of miRNAs in the ceRNA network.

ID	Description	Bg Ratio	*p* value	Adjusted *p*
hsa04921	Oxytocin signalling pathway	0.0212	1.3332E-08	2.9190E-06
hsa04261	Adrenergic signalling in cardiomyocytes	0.0208	6.2595E-07	6.5092E-05
hsa04024	cAMP signalling pathway	0.0273	8.9189E-07	6.5092E-05
hsa04510	Focal adhesion	0.0298	1.5422E-05	7.4010E-04
hsa04750	Inflammatory mediator regulation of TRP channels	0.0137	1.6901E-05	7.4010E-04
hsa04713	Circadian entrainment	0.0125	2.1580E-05	7.8746E-04
hsa04360	Axon guidance	0.0239	2.5931E-05	8.1108E-04
hsa04015	Rap1 signalling pathway	0.03	4.7767E-05	1.3073E-03
hsa05200	Pathways in cancer	0.055	6.2554E-05	1.5218E-03
hsa04611	Platelet activation	0.0165	7.0689E-05	1.5477E-03
hsa04010	MAPK signalling pathway	0.0381	1.1422E-04	2.2735E-03
hsa04724	Glutamatergic synapse	0.0149	1.3881E-04	2.3601E-03
hsa04725	Cholinergic synapse	0.0151	1.4013E-04	2.3601E-03
hsa05206	MicroRNAs in cancer	0.0193	2.6751E-04	4.1690E-03
hsa04728	Dopaminergic synapse	0.0165	2.8562E-04	4.1690E-03
hsa04925	Aldosterone synthesis and secretion	0.0108	3.4051E-04	4.6596E-03
hsa01522	Endocrine resistance	0.0132	3.9237E-04	4.6724E-03
hsa04722	Neurotrophin signalling pathway	0.0168	3.9400E-04	4.6724E-03
hsa04720	Long-term potentiation	0.0089	4.0547E-04	4.6724E-03
hsa04390	Hippo signalling pathway	0.0209	4.5090E-04	4.9362E-03
hsa04512	ECM–receptor interaction	0.0112	5.2201E-04	5.4425E-03
hsa04512	Wnt signalling pathway	0.0195	6.5195E-04	6.4883E-03
hsa04915	Oestrogen signalling pathway	0.0137	7.9656E-04	7.5828E-03
hsa04924	Renin secretion	0.0086	9.2945E-04	8.4792E-03
hsa04022	cGMP–PKG signalling pathway	0.0247	1.2704E-03	1.1126E-02
hsa04923	Regulation of lipolysis in adipocytes	0.0082	1.3531E-03	1.1192E-02
hsa05210	Colorectal cancer	0.009	1.4528E-03	1.1192E-02
hsa04014	Ras signalling pathway	0.0325	1.4684E-03	1.1192E-02
hsa04912	GnRH signalling pathway	0.0124	1.5787E-03	1.1192E-02
hsa04727	GABAergic synapse	0.0114	1.5828E-03	1.1192E-02
hsa04911	Insulin secretion	0.0116	1.5846E-03	1.1192E-02
hsa00512	Mucin type O-Glycan biosynthesis	0.0039	2.0450E-03	1.3992E-02
hsa04910	Insulin signalling pathway	0.0212	2.2097E-03	1.4661E-02
hsa00514	Other types of O-glycan biosynthesis	0.0042	2.3080E-03	1.4862E-02
hsa04012	ErbB signalling pathway	0.0121	3.0528E-03	1.8829E-02
hsa04270	Vascular smooth muscle contraction	0.017	3.0960E-03	1.8829E-02
hsa01212	Fatty acid metabolism	0.0068	4.0784E-03	2.3933E-02
hsa04020	Calcium signalling pathway	0.0302	4.2385E-03	2.3933E-02
hsa04930	Type II diabetes mellitus	0.0081	4.4096E-03	2.3933E-02
hsa04931	Insulin resistance	0.0158	4.4262E-03	2.3933E-02
hsa04971	Gastric acid secretion	0.0097	4.4817E-03	2.3933E-02
hsa04152	AMPK signalling pathway	0.018	4.7769E-03	2.4902E-02
hsa04211	Longevity regulating pathway	0.0135	5.2272E-03	2.6447E-02
hsa04916	Melanogenesis	0.0132	5.3149E-03	2.6447E-02
hsa04340	Hedgehog signalling pathway	0.0069	6.1564E-03	2.9698E-02
hsa04213	Longevity regulating pathway – multiple species	0.009	6.3540E-03	2.9698E-02
hsa05221	Acute myeloid leukaemia	0.0082	6.3751E-03	2.9698E-02
hsa04550	Signalling pathways regulating pluripotency of stem cells	0.0196	6.6773E-03	3.0458E-02
hsa05410	Hypertrophic cardiomyopathy (HCM)	0.0115	7.8953E-03	3.5279E-02
hsa05412	Arrhythmogenic right ventricular cardiomyopathy (ARVC)	0.0097	8.6718E-03	3.7274E-02
hsa04962	Vasopressin-regulated water reabsorption	0.006	8.6824E-03	3.7274E-02
hsa04144	Endocytosis	0.0376	9.4078E-03	3.9612E-02
hsa04068	FoxO signalling pathway	0.0201	9.9201E-03	4.0816E-02
hsa04350	TGF-beta signalling pathway	0.0119	1.0253E-02	4.0816E-02
hsa05222	Small cell lung cancer	0.0119	1.0253E-02	4.0816E-02
hsa01521	EGFR tyrosine kinase inhibitor resistance	0.0116	1.0447E-02	4.0847E-02
hsa00531	Glycosaminoglycan degradation	0.0026	1.1704E-02	4.4958E-02
hsa04723	Retrograde endocannabinoid signalling	0.0133	1.1967E-02	4.5173E-02
hsa04142	Lysosome	0.0175	1.2975E-02	4.8149E-02

KEGG, Kyoto Encyclopaedia of Genes and Genomes; ceRNA, competing endogenous RNA; miRNA, microRNA; Bg, background.

### Evaluation of Genomic Alterations, Drug Candidates/Repurposing and Pathways in SCLC ceRNA Network

In total, 50 of 58 mRNAs in the ceRNA network presented genomic alterations, with the percentage of genomic alterations ranging from 0.8% to 28% (**Table 2**). The drug–target gene pharmacogenomics analysis showed that three [colony-stimulating factor 3 receptor (CSF3R) (alterations range 1.3–7.0%, FC (in plasma cohort): -1.801, *p* = 2.63 x 10^-3^), acid alpha-glucosidase (GAA) (alterations range 1.3–3.0%, FC: -1.789 and *p* = 3.85 x 10^-2^), FGR proto-oncogene Src family tyrosine kinase (FGR) (alterations range 0–2.5%, FC: -1.488, *p* = 2.02 x 10^-2^)] of 50 mRNAs in the ceRNA network were identified as potential drug targets (**Tables 2** and **8**). CSF3R and GAA were identified as targets of FavId and Trastuzumab deruxtecan, respectively, while FGR was confirmed as a target of Dasatinib and Zanubrutinib (**Table 8**). Next, the pathway analysis found that CSF3R, GAA and FGR were annotated in the 13 pathways in the Genecards database (**Table 9**). The SCLC-associated pathways were further identified through a literature review (56–58). We concluded that CSF3R was involved in the autophagy pathway and GAA was involved in the glucose metabolism pathway, while these two pathways were involved in SCLC occurrence and progression from the literature (**Table 9**) (56–58).

**Table 8:** Potential drug candidates of mRNAs in the ceRNA networks in SCLC.

mRNAs	Drug candidate	Type*	Therapy*	Main roles*	Data resource
Colony-stimulating factor 3 receptor (CSF3R)	FavId	an active immunotherapy	Tumour therapy	based upon unique genetic information extracted from a patient’s tumour	https://go.drugbank.com/drugs/DB05249
	Pegfilgrastim	a recombinant human granulocyte colony stimulating factor	Adjuvant therapy	stimulate the production of neutrophils and prevent febrile neutropenia or infections after myelosuppressive chemotherapy	https://go.drugbank.com/drugs/DB00019
	Filgrastim	a form of recombinant human granulocyte colony stimulating factor	Adjuvant therapy	induce the production of granulocytes and lower infection risk after myelosuppressive therapy	https://go.drugbank.com/drugs/DB00099
	Lenograstim	a granulocyte colony-stimulating factor	Adjuvant therapy	reduce the duration of neutropenia in bone marrow transplant and cytotoxic chemotherapy, as well as mobilizing hematopoietic stem cells in healthy donors	https://go.drugbank.com/drugs/DB13144
	Lipegfilgrastim	a medication	Adjuvant therapy	reduce the duration of chemotherapy-induced neutropenia and incidence of febrile neutropenia in cytotoxic chemotherapy	https://go.drugbank.com/drugs/DB13200
Acid alpha-glucosidase (GAA)	Trastuzumab deruxtecan	an antibody	Tumour therapy	treat certain types of unresectable or metastatic HER-2 positive breast cancer	https://go.drugbank.com/drugs/DB14962
	Acarbose	an alpha-glucosidase inhibitor	Other therapy	adjunctly with diet and exercise for the management of glycaemic control in patients with type 2 diabetes mellitus.	https://go.drugbank.com/drugs/DB00284
	AT2220	pharmacological chaperones	Other therapy	increase GAA activity in cell lines derived from Pompe patients a n d in transfected cells expressing misfolded forms of GAA	https://go.drugbank.com/drugs/DB05200
	Miglitol	an oral alpha-glucosidase inhibitor	Other therapy	improve glycaemic control by delaying the digestion of carbohydrates	https://go.drugbank.com/drugs/DB00491
FGR Proto-Oncogene, Src Family Tyrosine Kinase (FGR)	Dasatinib	a tyrosine kinase inhibito	Tumour therapy	treat lymphoblastic or chronic myeloid leukaemia with resistance or intolerance to prior therapy	https://go.drugbank.com/drugs/DB01254
	Zanubrutinib	a kinase inhibitor	Tumour therapy	treat mantle cell lymphoma, a type of B-cell non-Hodgkin lymphoma, in adults who previously received therapy.	https://go.drugbank.com/drugs/DB015035
	Fostamatinib	a spleen tyrosine kinase inhibitor	Other therapy	treat chronic immune thrombocytopenia after attempting one other treatment.	https://go.drugbank.com/drugs/DB12010

ceRNA, competing endogenous RNA; SCLC, small cell lung cancer; HER-2, human epidermal growth factor receptor-2; *, the information is from Drugbank (https://go.drugbank.com/).

**Table 9:** Pathways of mRNAs in the ceRNA networks in SCLC.

mRNAs	Gene ontology (GO) based on molecular function	Pathways	Associated to SCLC pathway
Colony-stimulating factor 3 receptor (CSF3R)	Cytokine binding (GO:0019955)	Autophagy pathway	Güçlü E, et al. (56); Liu H, et al. (57)
	Cytokine receptor activity (GO:0004896)	Akt signalling	na
	Protein binding (GO:0005515)	PEDF-induced signalling	na
	Signalling receptor activity (GO:0038023)	Cytokine signalling in the immune system	na
	Granulocyte colony-stimulating factor binding (GO:0051916)	Hematopoietic cell lineage	na
Acid alpha-glucosidase (GAA)	Catalytic activity (GO:0003824)	Glucose metabolism	Yan X, et al. (58)
	Hydrolase activity, hydrolyzing O-glycosyl compounds (GO:0004553)	Innate immune system	na
	Alpha-1,4-glucosidase activity (GO:0004558)	Galactose metabolism	na
	Hydrolase activity (GO:0016787)	Metabolism	na
	Hydrolase activity, acting on glycosyl bonds (GO:0016798)	Lysosome	na
FGR Proto-Oncogene, Src Family Tyrosine Kinase (FGR)	Nucleotide binding (GO:0000166)	Innate immune system	na
	Phosphotyrosine residue binding (GO:0001784)	Platelet homeostasis	na
	Protein kinase activity (GO:0004672)	Tyrosine kinases/adaptors	na
	Protein tyrosine kinase activity (GO:0004713)	CCR5 pathway in macrophages	na
	Transmembrane receptor protein tyrosine kinase activity (GO:0004714)	Integrin pathway	na

ceRNA, competing endogenous RNA; SCLC, small cell lung cancer; Akt, protein kinase B; CCR5, chemokine-CC motif-receptor-5; GO, gene ontology; PEDF, pigment epithelium derived factor; na, not available.

### Identification of Multi-Omics Integration-Based Prioritisation of the ceRNA SCLC Network

**Figure 5:**
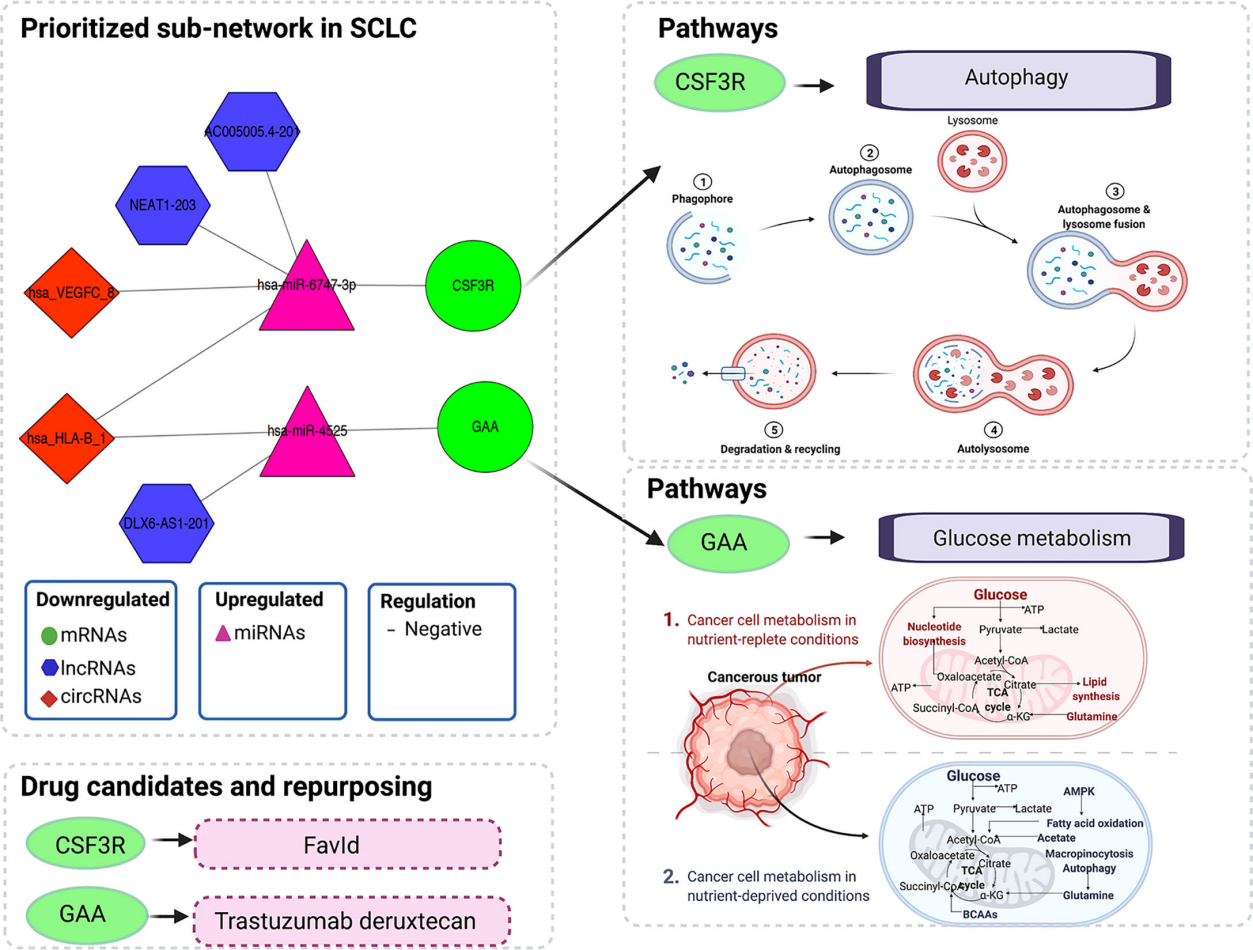
Illustration of multi-omics–based prioritisation of the ceRNA subnetwork, drug candidates and pathways. ATP, adenosine triphosphatase; AMPK, AMP-activated protein kinase; BCAAs, branched-chain amino acids; CoA, coenzyme A; ceRNA, competitive endogenous; RNA; circRNA, circular RNA; CSF3R, colony-stimulating factor 3 receptor; GAA, acid alpha-glucosidase; lncRNA, long noncoding RNA; miRNA, microRNA; mRNA, messenger RNA; SCLC, small cell lung cancer; TCA, tricarboxylic acid.

The multi-omics integration-based prioritisation of the ceRNA regulatory network in SCLC consisted of two mRNAs, two miRNAs, three lncRNAs and two circRNAs (**Figure 5**). In this ceRNA network, the expression levels of mRNAs (CSF3R/GAA), lncRNAs (AC005005.4-201/DLX6-AS1-201/NEAT1-203) and circRNAs (hsa_HLA-B_1/hsa_VEGFC_8) decreased in SCLC, while the expression levels of miRNAs (hsa-miR-4525/hsa-miR-6747-3p) increased in SCLC. The primary regulatory axes in the ceRNA network were identified as follows: 1) lncRNA-miRNA-mRNA: AC005005.4-201/NEAT1-203-hsa-miR-6747-3p-CSF3R and DLX6-AS1-201-hsa-miR-4525-GAA; and 2) circRNA-miRNA-mRNA: hsa_HLA-B_1/hsa_VEGFC_8-hsa-miR-6747-3p-CSF3R and hsa_HLA-B_1-hsa-miR-4525-GAA (**Figure 5**). Thus, lncRNAs (lncRNA-AC005005.4-201 and NEAT1-203) and circRNAs (circRNA-hsa_HLA-B_1 and hsa_VEGFC_8) may regulate the inhibited effects of hsa-miR-6747-3p for CSF3R expression in SCLC, and lncRNA-DLX6-AS1-201 or circRNA-hsa_HLA-B_1 may neutralise the negative regulation of hsa-miR-4525 for GAA in SCLC.

## Discussion

Here, we integrated our own omics data (transcriptome and regulome) and public omics data (genome and pharmacogenome) to elucidate the multi-omics integration-based prioritisation of ceRNA-mediated network characteristics, pathways and drug candidates in SCLC. The prioritisation of the SCLC ceRNA regulatory network consisted of two mRNAs (CSF3R/GAA), two miRNAs (hsa-miR-4525/hsa-miR-6747-3p), three lncRNAs (AC005005.4-201/DLX6-AS1-201/NEAT1-203) and two circRNAs (hsa_HLA-B_1/hsa_VEGFC_8). The expression levels of mRNAs, lncRNAs and circRNAs decreased in SCLC, while the expression levels of miRNAs increased in SCLC. In addition, lncRNAs (lncRNA-AC005005.4-201 and NEAT1-203) and circRNAs (circRNA-hsa_HLA-B_1 and hsa_VEGFC_8) may regulate the inhibited effects of hsa-miR-6747-3p for CSF3R expression in SCLC, and lncRNA-DLX6-AS1-201 or circRNA-hsa_HLA-B_1 may neutralise the negative regulation of hsa-miR-4525 related to GAA in SCLC. The pharmacogenomics analysis identified CSF3R and GAA as targets of FavId and Trastuzumab deruxtecan, respectively. The SCLC-associated pathway analysis revealed that CSF3R was involved in the autophagy pathway, while GAA was involved in the glucose metabolism pathway. These findings may contribute to understanding the molecular pathogenesis of SCLC, supporting the development of novel diagnostics and therapeutic compounds for SCLC patients in clinical settings.

In this study, we first reported the multi-omics integration-based prioritisation of the lncRNA/circRNA-miRNA-mRNA ceRNA disease network, as well as the molecular characteristics and drug candidates or repurposed drugs in SCLC. The ceRNA is a layer of gene regulation in diseases, and the transcripts can regulate each other at the post-transcription level by competing for shared miRNAs (12, 16, 17). Here, we found that two lncRNAs (lncRNA-AC005005.4-201 and NEAT1-203) and two circRNAs (circRNA-hsa_HLA-B_1 and hsa_VEGFC_8) may regulate the inhibiting effects of hsa-miR-6747-3p for CSF3R expression, while lncRNA-DLX6-AS1-201 or circRNA-hsa_HLA-B_1 may neutralise the negative regulation of hsa-miR-4525 for GAA. Consistent with our findings for dysregulated lncRNAs in SCLC, previous studies found that lncRNAs DLX6-AS1 and NEAT1 were significantly dysregulated in non-SCLC, gastric cancer and pancreatic cancer (59–62). Specifically, upregulated DLX6-AS1 in gastric cancer tissue associated with distant metastasis and a poor clinical prognosis, while siRNA-DLX6-AS1 may inhibit gastric cancer cell proliferation, migration, invasion and the epithelial–mesenchymal transition *in vitro* (18). In addition, our study identified the regulatory axis in lncRNA-DLX6-AS1-201/hsa-miR-4525/GAA, which associated with the glucose metabolism pathway in SCLC. Interestingly, Qian et al. reported that sh-DLX6-AS1 may modulate glucose metabolism and cell growth *via* miR-4290/3-phosphoinositide-dependent protein kinase 1 in gastric cancer cells (63). Considering the role of DLX6-AS1 in glucose metabolism, we inferred that DLX6-AS1 could affect the occurrence and progression of SCLC *via* glucose metabolism through modulating hsa-miR-4525/GAA in SCLC. Similar to the other dysregulated lncRNA reports (59–62), Xu et al. found that lncRNA-NEAT1 may promote gastric cancer angiogenesis by enhancing the proliferation, migration and tube formation ability of endothelial cells through the miR-17-5p/transforming growth factor-β receptor 2 (TGFβR2) pathway (61), while lncRNA-NEAT1 may play a vital role in tumorigenesis and the development of SCLC through the hsa-miR-6747-3p/CSF3R axis. Importantly, in addition to lncRNA-DLX6-AS1 and NEAT1, we are the first to report another potential regulatory axis of ceRNA, while the regulatory mechanisms require further exploration through *in vivo* and *in vitro* studies. Our findings, however, suggest that the promising lncRNA/circRNA-miRNA-mRNA ceRNA regulatory characteristics in SCLC may provide new potential mechanisms and therapeutic targets.

To the best of our knowledge, this is also the first study to investigate the roles of CSF3R and GAA in the SCLC ceRNA regulation networks, pathways and drug candidates. CSF3R is a type 1 cytokine receptor, encoding the receptor for granulocyte colony-stimulating factor (G-CSF) and playing a crucial role in granulocyte proliferation and differentiation (64, 65). The altered CSF3R expression or activating heterozygous variants in CSF3R have been identified as risk factors in the development of multiple malignancies, such as colorectal cancer, myeloid malignancies and lymphoid malignancies (65–67). This is particularly the case for mutations in CSF3R commonly present in chronic neutrophilic leukaemia or atypical chronic myeloid leukaemia (68). Given the roles of CSF3R reported in chronic neutrophilic leukaemia or atypical chronic myeloid leukaemia (66, 68), our findings suggest that CSF3R might play a pivotal role in the occurrence and development of SCLC. Furthermore, our results suggest that CSF3R might modulate the autophagy pathway, which associated with SCLC (57, 58). The functions of autophagy in cancer may involve an anticancer or a cancer effect (69). Previous studies suggested that a hypoxia-HIF1A-AS2-autophagy interaction may play a role in drug sensitivity in SCLC, while a high expression of secreted phosphoprotein 1 (SPP1) inhibited autophagy and apoptosis, promoting the development of SCLC (57, 58). In addition, Rupniewska et al. found that SCLC cells may be more sensitive to autophagy inhibitors (70). In our study, CSF3R was identified as the potential drug target of FavId. FavId is an active immunotherapy with stimulating tumour-specific T cells and humoural immunity (71, 72). Alissafi et al. reported that autophagy-deficient therapy exhibited a mediated suppression of antitumour immunity *via* the efficient activation of tumour-specific CD4+ T cells (73), which was consistent with the mechanism of FavId in a tumour. Thus, our results suggest that genetic alterations or an altered expression of CSF3R may serve as a risk factor in SCLC development and associate with the autophagy pathway, while FavId could serve as a potential drug therapy through the CSF3R target to treat SCLC, even though additional *in vivo* or *in vitro* studies are needed to clarify these associations in SCLC. GAA, as one of the lysosomal enzymes, was the other key gene in our study. This is the first study to find that GAA might participant in the occurrence and development of SCLC *via* glucose metabolism. Similarly, Hamura et al. reported that the modulation of GAA could affect cell proliferation and apoptosis and manipulate chemoresistance in pancreatic cancer cells *via* malfunctional mitochondria (74). The dysregulated metabolism of glucose in mitochondria is known as an adverse microenvironment in solid tumours, referred to as the Warburg effect, including glucose deprivation and lactic acidosis, potentially resulting in an elevated glycolytic activity in tumour cells (75–78). Yan et al. showed that glucose metabolic reprogramming improves SCLC cell proliferation and metastasis, suggesting it could be a potential regulatory strategy interfering with glucose metabolism in SCLC (56). Considering the function of GAA, which catalyses the production of glucose from glycogen in lysosomes, altering the GAA expression or genetic status could inhibit tumorigenesis in SCLC through the lysosome pathway (56, 74–78). Interestingly, the DrugBank analysis showed that the drug targeting GAA was Trastuzumab-deruxtecan. Trastuzumab-deruxtecan is primarily used for patients with human epidermal growth factor receptor 2 (HER2)–mutant tumours including non-SCLC and in the absence of SCLC (79–81). Upon binding to HER2, Trastuzumab-deruxtecan disrupts the HER2 signalling, undergoes internalisation and intracellular linker cleavage by lysosomal enzymes and ultimately causes DNA damage and apoptotic cell death (80). In addition, Martinho et al. found that the inhibitors of the HER family (mainly HER2) reduced cervical cancer aggressiveness by blocking glucose metabolism (82). Combined with the roles of the glucose metabolism pathway in SCLC and the antitumour roles of Trastuzumab-deruxtecan *via* the glucose metabolism pathway, our findings suggest that Trastuzumab-deruxtecan may be a promising drug candidate *via* GAA in SCLC through the glucose metabolism pathway. However, further *in vivo* or *in vitro* studies are needed to clarify these promising drug candidates’ ability to treat SCLC.

The strength of this study is our use of network-based multi-omics integration to prioritise ceRNA characteristics and drug candidates in SCLC from two well-characterised study cohorts, including newly tested whole-transcriptome sequencing data in the SCLC study, and the data were uploaded to a public platform [the Sequence Read Archive (SRA) database]. In addition to these strengths, we also note several limitations. First, our study included our own omics data and public data. In addition, the relatively small size of our cohort represents a limitation to our findings, although the results of the mRNA study were validated in a relatively large cohort. Second, the ceRNA characteristics and drug candidates and repurposing are quite promising, although further mechanistic studies from cells and animal models, as well as clinical validation studies, are needed. In addition, we performed no survival analysis in this study, since no available and suitable survival data were obtained from public databases, including the Cancer Genome Atlas (TCGA) and Kaplan–Meier plotter databases. Finally, the survival data in our SCLC plasma cohort were incapable of producing useful results for the prognostic analysis given the relatively small sample sizes and quite limited follow-up time.

In conclusion, we report primary findings related to a multi-omics integration-based prioritisation of the lncRNA/circRNA-miRNA-mRNA ceRNA regulatory network, pathways and promising drug candidates in SCLC. These findings indicate novel, potential diagnostic and therapeutic targets in SCLC.

## Data Availability Statement

The datasets presented in this study can be found in online repositories. The names of the repository/repositories and accession number(s) can be found in the article/**Supplementary Material**.

## Ethics Statement

This study received ethical approval from the Ethics Committee of the Gansu Provincial Hospital, China (27 July 2020, No. 2020-183). The patients/participants provided their written informed consent to participate in this study.

## Author Contributions

W-DH, MZ, X-JW and JG contributed to the design of the study. X-JW and W-DH performed the sample collection, analysis and downloaded the data. X-JW and JG contributed to the data analysis and to writing the manuscript. W-DH, MZ, QY, X-JW and JG revised the manuscript. All authors approved the final version of the manuscript.

## Funding

This study was supported by the Science–Technology Foundation for Young Scientist of the Gansu Province of China (Grant no.18JR3RA059), the Science–Technology Foundation for Scientists of the Gansu Province of China (Grant no.21JR7RA595), the Science–Technology Foundation for Lanzhou City of China (Grant no.2018-4-65) and the Scientists Fund of the Gansu Provincial Hospital of China (Grant no.18GSS4-25). Jing Gao was also supported by the Swedish Heart–Lung Foundation, the Swedish Asthma and Allergy Foundation, the Sigrid Jusélius Foundation and the Väinö and Laina Kivi Foundation.

## Conflict of Interest

The authors declare that the research was conducted in the absence of any commercial or financial relationships that could be construed as a potential conflict of interest.

## Publisher’s Note

All claims expressed in this article are solely those of the authors and do not necessarily represent those of their affiliated organizations, or those of the publisher, the editors and the reviewers. Any product that may be evaluated in this article, or claim that may be made by its manufacturer, is not guaranteed or endorsed by the publisher.

## Supplementary Material

Supplementary Table 1 The lncRNAs, miRNAs and mRNAs in the lncRNA-miRNA-mRNA ceRNA network.

Supplementary Table 2 The circRNAs, miRNAs and mRNAs in the circRNA-miRNA-mRNA ceRNA network.

Supplementary Table 3 Differentially expressed levels of 301 lncRNAs in the ceRNA network.

Supplementary Table 4 Differentially expressed levels of 16 circRNAs in the ceRNA network.

Supplementary Table 5 Differentially expressed levels of 24 miRNAs in the ceRNA network.

Supplemental File 1 The whole-transcriptome sequencing process.

Supplemental File 2 Data cleaning process.
