# Supplementary material for: Multi-Omics Integration-Based Prioritisation of Competing Endogenous RNA Regulation Networks in Small Cell Lung Cancer: Molecular Characteristics and Drug Candidates
Source: Front Oncol. 2022 Jul 4;12:904865. doi: 10.3389/fonc.2022.904865 (PMC9291301; doi:10.3389/fonc.2022.904865)
Supplement: Supplementary Table 1 — The lncRNAs, miRNAs and mRNAs in the lncRNA-miRNA-mRNA ceRNA network. [file DataSheet_1.docx]

Supplementary Material

# Supplementary Table

**Table 1.** The lncRNAs, miRNAs and mRNAs in the lncRNA-miRNA-mRNA ceRNA network.

| Type | Regulated | ID | Total number |
| --- | --- | --- | --- |
| lncRNA | Upregulated | MSTRG.347501.1, MSTRG.300186.90, MSTRG.857748.21, MSTRG.850408.1, MSTRG.60114.9, MSTRG.60114.11, MSTRG.542040.1, MSTRG.432681.1, MSTRG.429372.33, MSTRG.228421.22, MSTRG.177730.44, MSTRG.161919.4, MSTRG.1536691.2, MSTRG.1496194.103, MSTRG.1466666.12, MSTRG.1218681.17, MSTRG.1179892.96, MSTRG.111865.2, MSTRG.1105296.2, MSTRG.1093798.5, MSTRG.1003449.6, MSTRG.967786.8, MSTRG.967786.3, MSTRG.779771.6, MSTRG.713518.27, MSTRG.68267.23, MSTRG.1513646.4, MSTRG.1208646.23, MSTRG.1187818.28, MSTRG.1066945.21, AC103718.1201, MSTRG.673810.71, MSTRG.876263.22, MSTRG.429372.30, MSTRG.659351.12, MSTRG.1010132.3, MSTRG.655305.2, MSTRG.578240.3, MSTRG.568285.48, MEG3-210 | 40 |
|  | Downregulated | MSTRG.851945.19, MSTRG.645614.1, MSTRG.235213.57, MSTRG.1394234.18, MSTRG.7016.17, MSTRG.58118.2, MSTRG.554733.51, MSTRG.539847.8, MSTRG.538377.22, MSTRG.255509.39, MSTRG.1377135.4, MSTRG.1357008.75, MSTRG.190512.30, MSTRG.1537320.13, MSTRG.905566.229, MSTRG.875093.37, MSTRG.827381.22, MSTRG.705165.38, MSTRG.493398.29, MSTRG.247052.141, MSTRG.1314749.102, AL049840.1-201, MSTRG.1539120.6, MSTRG.1319497.22, MSTRG.1179892.148, MSTRG.705165.8, MSTRG.794456.24, MSTRG.72551.20, MSTRG.705165.78, MSTRG.513682.45, MSTRG.234429.7, MSTRG.163201.39, MSTRG.1331160.48, MSTRG.980155.15, MSTRG.95360.15, MSTRG.893754.15, MSTRG.844972.3, MSTRG.80030.63, MSTRG.752109.47, MSTRG.685856.25, MSTRG.685518.32, MSTRG.6687.20, MSTRG.595683.34, MSTRG.561854.49, MSTRG.561532.38, MSTRG.545186.4, MSTRG.494241.37, MSTRG.429372.8, MSTRG.429372.19, MSTRG.429372.18, MSTRG.429370.3, MSTRG.349808.8, MSTRG.260129.62, MSTRG.247052.131, MSTRG.163201.87 MSTRG.1373338.172, MSTRG.1373338.124, MSTRG.1363133.8, MSTRG.13457.70, MSTRG.1283690.82, MSTRG.1222550.19, MSTRG.1201985.2, MSTRG.115720.42, MSTRG.1067349.42, MSTRG.1066722.55, MSTRG.1044601.12, AP000347.2-202, MSTRG.981882.51, MSTRG.62509.8, MSTRG.539573.10, MSTRG.4701.39, MSTRG.876829.7, MSTRG.190771.21, MSTRG.147703.2, MSTRG.810520.26, MSTRG.703759.4, MSTRG.668360.15, MSTRG.628968.1, MSTRG.1372519.1, MSTRG.1341188.2, MSTRG.1276893.16, MSTRG.1202417.42, MSTRG.1196158.104, MSTRG.1068754.98, MSTRG.1068754.72, MSTRG.1068754.64, MSTRG.733623.26, MSTRG.556836.57, MSTRG.46411.55, MSTRG.1068754.6, AC005358.2-201, MSTRG.177832.10, MSTRG.783530.106, MSTRG.876829.31, MSTRG.788069.2, MSTRG.432644.45, MSTRG.404975.1, MSTRG.1179892.56, MSTRG.356566.3, MSTRG.1319497.23, MSTRG.979666.27, MSTRG.891911.1, MSTRG.880192.55, MSTRG.874430.72, MSTRG.783384.58, MSTRG.700846.24, MSTRG.580214.74, MSTRG.507925.17, MSTRG.486582.32, MSTRG.24423.3, MSTRG.235491.88, MSTRG.175045.1, MSTRG.153922.7, MSTRG.1518513.56, MSTRG.1428590.84, MSTRG.1336447.5, MSTRG.1216919.21, MSTRG.1202481.1, MSTRG.706722.47, MSTRG.66466.76, MSTRG.570783.71, MSTRG.545824.15, MSTRG.463852.5, MSTRG.14279.24, MSTRG.1184274.14, MSTRG.354530.20, MSTRG.1314749.47, AL160408.3-201, MSTRG.946394.5, MSTRG.891911.3, MSTRG.880388.22, MSTRG.876264.6, MSTRG.743725.37, MSTRG.743725.35, MSTRG.676775.13, MSTRG.674800.31, MSTRG.661988.85, MSTRG.64895.80, MSTRG.641478.3, MSTRG.610942.62, MSTRG.609850.62, MSTRG.58408.5, MSTRG.561154.51, MSTRG.550192.2, MSTRG.490497.6, MSTRG.481175.25, MSTRG.429372.17, MSTRG.429370.42, MSTRG.429370.40, MSTRG.429370.25, MSTRG.347155.36, MSTRG.20799.28, MSTRG.204006.25, MSTRG.1412398.58, MSTRG.1341295.2, MSTRG.1336322.32, MSTRG.1286937.8, MSTRG.1262019.32, MSTRG.1222025.71, MSTRG.1215689.7, MSTRG.1201972.2, MSTRG.1177863.16, MSTRG.115589.81, MSTRG.1149269.23, MSTRG.1048212.103, MSTRG.1024651.65, MSTRG.937216.5, MSTRG.766467.123, MSTRG.738437.1, MSTRG.673810.17, MSTRG.571069.25, MSTRG.542002.161, MSTRG.459672.4, MSTRG.429370.35, MSTRG.429370.34, MSTRG.33837.21, MSTRG.305261.15, MSTRG.296917.28, MSTRG.267110.2, MSTRG.1403237.30, MSTRG.1363133.9, MSTRG.1283690.86, MSTRG.1228729.48, MSTRG.1214301.25, MSTRG.1176055.2, MSTRG.1119798.39, MSTRG.1048212.100, MSTRG.439169.16, MSTRG.855649.68, MSTRG.634792.46, MSTRG.631640.7, MSTRG.627239.24, MSTRG.500136.3, MSTRG.450395.170, MSTRG.339846.13, MSTRG.276053.3, MSTRG.1073576.1, MSTRG.668360.21, MSTRG.667092.36, MSTRG.161783.1, MSTRG.1365204.12, MSTRG.705569.54, MSTRG.494241.42, MSTRG.229691.13, MSTRG.1387045.2, NEAT1-203, MSTRG.545824.44, MSTRG.850412.2, MSTRG.848607.16, MSTRG.1447356.5, DLX6-AS1-201, MSTRG.865821.1, MSTRG.1494844.8, MSTRG.1494844.10, MSTRG.266589.1, MSTRG.705165.76, MSTRG.661988.71, MSTRG.1568573.9, MSTRG.1568573.6, MSTRG.1562716.5, MSTRG.1460840.48, MSTRG.1388138.12, MSTRG.1360566.57, MSTRG.1291133.29, MSTRG.650258.2,  MSTRG.980678.9, MSTRG.791149.2, MSTRG.631387.1, MSTRG.331514.143, MSTRG.284810.2, MSTRG.1291133.25, HCP5-204, MSTRG.818647.55, MSTRG.62228.21, MSTRG.1435009.17, MSTRG.1031848.3, MSTRG.1028640.22, MSTRG.786739.4, MSTRG.676524.26, MSTRG.1357008.140, MSTRG.1216919.24, MSTRG.666747.70, MSTRG.494241.44, MSTRG.673810.97, MSTRG.668524.12, MSTRG.561410.61, MSTRG.481547.64, MSTRG.478521.2, MSTRG.247052.121, MSTRG.1474777.60, MSTRG.1456373.57, MSTRG.1263035.20, MSTRG.73810.50, MSTRG.1139922.2, MSTRG.844962.27, MSTRG.409396.48, MSTRG.200642.18, AC005005.4-201, MSTRG.484748.6, MSTRG.1281040.29 | 261 |
| miRNA | Upregulated | novel_miR_1016, novel_miR_93, novel_miR_821, novel_miR_76, novel_miR_728, novel_miR_662, novel_miR_426, novel_miR_363, novel_miR_186, hsa-miR-6850-5p, hsa-miR-6747-3p, hsa-miR-4732-3p, hsa-miR-4525, hsa-miR-4467, hsa-miR-3940-3p, hsa-miR-3620-3p, hsa-miR-216a-3p, hsa-miR-1303, hsa-miR-1269b | 19 |
|  | Downregulated | novel_miR_936, novel_miR_747, novel_miR_746, hsa-miR-652-5p | 4 |
| mRNA | Upregulated | ATRNL1, ZNF704, NOVA1, GREM1 | 4 |
|  | Downregulated | CFD, AKNA, EMILIN2, ADAMTSL4, PSD4, RHBDF2, SLC27A1, MYO15B, DNAH1, ATG16L2, ABCA2, ADCY7, STAB1, DAPK1, NLRP12, XAF1, FCN1, PRF1, CD300E, PLCB2, IRF1, FGR, CD93, ADAM15, SYNE1, HERC3, CSF3R, PARP10, IL10RA, MYO1G, MEFV, ITGAL, GAA, MYO1F, ITGAX, FOSB, EGR1, AHNAK, TCIRG1, NOTCH1, KLF6, FGD2, TTC7A, PLXNB2, GIMAP8, CIITA, CD244, ARHGAP4, RNF213, ITGB2, APLP2, PARP14, NFAM1 | 53 |

Note: CeRNA, competitive endogenous RNA; lncRNA, long noncoding RNA; miRNA, MicroRNA; mRNA, messenger RNA.

**Table 2.** The circRNAs, miRNAs and mRNAs in circRNA-miRNA-mRNA ceRNA network.

| **Type** | **Regulated** | **ID** | **Total number** |
| --- | --- | --- | --- |
| circRNA | Upregulated | hsa_intergenic_167, hsa_CDK8_1, hsa_PFKFB4_1, hsa_NSMCE2_2, hsa_RABGAP1_20 | 5 |
|  | Downregulated | hsa_RUFY1_1, hsa_intergenic_20, hsa_intergenic_526, hsa_intergenic_570, hsa_REV3L_16, hsa_CD226_6, hsa_SCFD2_6, hsa_intergenic_234, hsa_VEGFC_8, hsa_intergenic_773, hsa_HLA-B_1 | 11 |
| miRNA | Upregulated | novel_miR_1016, novel_miR_93, novel_miR_821, novel_miR_76, novel_miR_728, novel_miR_662, novel_miR_426, novel_miR_363, novel_miR_186, hsa-miR-6747-3p, hsa-miR-4525, hsa-miR-3620-3p, hsa-miR-216a-3p, hsa-miR-1303, hsa-miR-1269b, hsa-miR-1180-3p | 16 |
|  | Downregulated | novel_miR_936, novel_miR_747, novel_miR_746 | 3 |
| mRNA | Upregulated | NOVA1, GREM1, ZNF704, ATRNL1 | 4 |
|  | Downregulated | CFD, AKNA, EMILIN2, PSD4, MYO15B, ATG16L2, ADAMTSL4, ADCY7, RHBDF2, ARHGEF1, MYO1F, NLRP12, XAF1, FCN1, PRF1, CD300E, PLCB2, PARP10, IRF1, CD93, HERC3, CSF3R, MYO1G, NOTCH1, MEFV, ITGAL, GAA, TTC7A, GIMAP8, CD244, ARHGAP4, RNF213, ITGB2, APLP2, PARP14, SYNE1, STAB1, NFAM1, PLXNB2, FGR, FGD2, DAPK1, CIITA | 43 |

Note: CeRNA, competitive endogenous RNA; circRNA, circular RNAs; miRNA, MicroRNA; mRNA, messenger RNA.

**Table 3.** Differentially expressed levels of 301 lncRNAs in the ceRNA network.

| **LncRNAs Symbol** | ***p* value** | **log2FC** | **Regulated** |
| --- | --- | --- | --- |
| MSTRG.981882.51 | 0.031913 | Inf | up |
| MSTRG.4701.39 | 0.036684 | Inf | up |
| MSTRG.147703.2 | 0.014347 | Inf | up |
| MSTRG.628968.1 | 0.043065 | Inf | up |
| MSTRG.1068754.64 | 0.002265 | 5.847481 | up |
| MSTRG.556836.57 | 0.002467 | 7.71969 | up |
| MSTRG.46411.55 | 0.00148 | Inf | up |
| MSTRG.1068754.6 | 0.011165 | 5.425562 | up |
| AC005358.2-201 | 0.043383 | Inf | up |
| MSTRG.404975.1 | 0.044655 | Inf | up |
| MSTRG.356566.3 | 0.025507 | Inf | up |
| MSTRG.783384.58 | 0.046044 | 2.692697 | up |
| MSTRG.580214.74 | 0.0044 | Inf | up |
| MSTRG.486582.32 | 0.025567 | Inf | up |
| MSTRG.1428590.84 | 0.00307 | Inf | up |
| MSTRG.1202481.1 | 0.027211 | Inf | up |
| MSTRG.570783.71 | 0.015505 | 4.66992 | up |
| MSTRG.545824.15 | 0.041591 | 6.540411 | up |
| MSTRG.463852.5 | 0.011021 | 5.017106 | up |
| MSTRG.14279.24 | 0.025668 | Inf | up |
| AL160408.3-201 | 0.025909 | Inf | up |
| MSTRG.946394.5 | 0.000948 | Inf | up |
| MSTRG.876264.6 | 0.002467 | 8.177292 | up |
| MSTRG.743725.35 | 2.25E-06 | Inf | up |
| MSTRG.58408.5 | 0.000348 | Inf | up |
| MSTRG.550192.2 | 0.015807 | 3.216529 | up |
| MSTRG.429370.25 | 0.030465 | 6.07276 | up |
| MSTRG.347155.36 | 0.031152 | Inf | up |
| MSTRG.204006.25 | 0.005459 | Inf | up |
| MSTRG.1412398.58 | 0.038204 | Inf | up |
| MSTRG.1286937.8 | 0.00405 | Inf | up |
| MSTRG.1262019.32 | 0.000205 | Inf | up |
| MSTRG.1215689.7 | 0.035382 | 4.982036 | up |
| MSTRG.1177863.16 | 0.014599 | 4.772332 | up |
| MSTRG.1149269.23 | 0.004243 | 5.959942 | up |
| MSTRG.1048212.103 | 0.032729 | Inf | up |
| MSTRG.1024651.65 | 0.036162 | Inf | up |
| MSTRG.738437.1 | 0.042031 | Inf | up |
| MSTRG.459672.4 | 0.006485 | Inf | up |
| MSTRG.429370.35 | 0.035308 | Inf | up |
| MSTRG.267110.2 | 0.033341 | Inf | up |
| MSTRG.1403237.30 | 0.003356 | Inf | up |
| MSTRG.1363133.9 | 0.014674 | Inf | up |
| MSTRG.1119798.39 | 0.01045 | Inf | up |
| MSTRG.855649.68 | 0.006681 | 7.887261 | up |
| MSTRG.500136.3 | 0.005109 | 5.223886 | up |
| MSTRG.276053.3 | 0.022422 | Inf | up |
| MSTRG.1073576.1 | 0.035584 | 3.521206 | up |
| MSTRG.668360.21 | 0.039464 | 3.896291 | up |
| MSTRG.1365204.12 | 0.024586 | 3.610915 | up |
| MSTRG.229691.13 | 0.013697 | 3.52832 | up |
| MSTRG.1387045.2 | 0.020818 | Inf | up |
| MSTRG.545824.44 | 0.025744 | 3.650057 | up |
| MSTRG.850412.2 | 0.046016 | Inf | up |
| DLX6-AS1-201 | 0.032065 | Inf | up |
| MSTRG.1291133.29 | 0.018062 | Inf | up |
| MSTRG.980678.9 | 0.023212 | Inf | up |
| MSTRG.791149.2 | 0.01066 | Inf | up |
| MSTRG.284810.2 | 0.036486 | 4.485194 | up |
| MSTRG.1291133.25 | 0.002874 | Inf | up |
| MSTRG.786739.4 | 0.034223 | Inf | up |
| MSTRG.666747.70 | 0.020311 | Inf | up |
| MSTRG.478521.2 | 0.000799 | Inf | up |
| MSTRG.1139922.2 | 0.017467 | Inf | up |
| MSTRG.844962.27 | 0.039874 | Inf | up |
| AC005005.4-201 | 0.045135 | 4.552049 | up |
| MSTRG.347501.1 | 0.044079 | Inf | up |
| MSTRG.300186.90 | 0.029712 | Inf | up |
| MSTRG.857748.21 | 0.009304 | Inf | up |
| MSTRG.850408.1 | 0.001229 | Inf | up |
| MSTRG.60114.9 | 0.044137 | 3.720551 | up |
| MSTRG.60114.11 | 0.032797 | 4.83991 | up |
| MSTRG.542040.1 | 0.016463 | Inf | up |
| MSTRG.432681.1 | 0.043498 | 3.661386 | up |
| MSTRG.429372.33 | 0.044441 | 6.636175 | up |
| MSTRG.228421.22 | 0.032664 | Inf | up |
| MSTRG.177730.44 | 0.008767 | Inf | up |
| MSTRG.161919.4 | 0.03977 | Inf | up |
| MSTRG.1536691.2 | 0.033984 | Inf | up |
| MSTRG.1496194.103 | 0.032593 | Inf | up |
| MSTRG.1466666.12 | 0.030739 | Inf | up |
| MSTRG.1218681.17 | 0.04269 | 3.446193 | up |
| MSTRG.1179892.96 | 0.000505 | Inf | up |
| MSTRG.111865.2 | 0.00099 | 5.673858 | up |
| MSTRG.1105296.2 | 0.046526 | 5.504911 | up |
| MSTRG.1003449.6 | 8.6E-06 | Inf | up |
| MSTRG.967786.8 | 0.048602 | Inf | up |
| MSTRG.967786.3 | 0.029666 | Inf | up |
| MSTRG.779771.6 | 0.007094 | Inf | up |
| MSTRG.713518.27 | 0.007721 | Inf | up |
| MSTRG.68267.23 | 0.001999 | Inf | up |
| MSTRG.1208646.23 | 0.003207 | 7.424476 | up |
| MSTRG.1187818.28 | 0.04047 | Inf | up |
| MSTRG.1066945.21 | 0.02697 | Inf | up |
| MEG3-210 | 0.008842 | Inf | up |
| AC103718.1-201 | 0.042539 | Inf | up |
| MSTRG.673810.71 | 0.005812 | Inf | up |
| MSTRG.876263.22 | 0.03058 | 7.393478 | up |
| MSTRG.429372.30 | 0.041056 | Inf | up |
| MSTRG.659351.12 | 0.026201 | Inf | up |
| MSTRG.1010132.3 | 0.029177 | 5.66927 | up |
| MSTRG.655305.2 | 0.048901 | Inf | up |
| MSTRG.578240.3 | 0.023158 | Inf | up |
| MSTRG.568285.48 | 0.038378 | 3.557759 | up |
| MSTRG.851945.19 | 0.028414 | -Inf | down |
| MSTRG.645614.1 | 0.016709 | -Inf | down |
| MSTRG.235213.57 | 0.014898 | -Inf | down |
| MSTRG.1394234.18 | 0.04881 | -2.08913 | down |
| MSTRG.7016.17 | 0.031803 | -Inf | down |
| MSTRG.58118.2 | 0.012809 | -Inf | down |
| MSTRG.554733.51 | 0.025561 | -2.83879 | down |
| MSTRG.539847.8 | 0.00311 | -Inf | down |
| MSTRG.538377.22 | 0.034916 | -Inf | down |
| MSTRG.255509.39 | 0.006328 | -4.15468 | down |
| MSTRG.1377135.4 | 0.037623 | -3.33272 | down |
| MSTRG.1357008.75 | 0.024895 | -4.1717 | down |
| MSTRG.190512.30 | 0.045651 | -7.05402 | down |
| MSTRG.1537320.13 | 0.037535 | -Inf | down |
| MSTRG.905566.229 | 0.033412 | -Inf | down |
| MSTRG.875093.37 | 0.038113 | -Inf | down |
| MSTRG.827381.22 | 0.017096 | -12.813 | down |
| MSTRG.705165.38 | 0.013985 | -7.74595 | down |
| MSTRG.493398.29 | 0.000388 | -7.21131 | down |
| MSTRG.247052.141 | 0.036969 | -Inf | down |
| MSTRG.1314749.102 | 0.003165 | -Inf | down |
| AL049840.1-201 | 0.010648 | -Inf | down |
| MSTRG.1539120.6 | 0.0415 | -2.44952 | down |
| MSTRG.1319497.22 | 0.006207 | -4.15326 | down |
| MSTRG.1179892.148 | 0.041601 | -4.34738 | down |
| MSTRG.705165.8 | 0.000967 | -Inf | down |
| MSTRG.794456.24 | 0.034635 | -Inf | down |
| MSTRG.72551.20 | 0.021557 | -Inf | down |
| MSTRG.705165.78 | 0.032906 | -Inf | down |
| MSTRG.513682.45 | 0.007936 | -Inf | down |
| MSTRG.234429.7 | 0.042001 | -2.02229 | down |
| MSTRG.163201.39 | 5.57E-07 | -Inf | down |
| MSTRG.1331160.48 | 0.002881 | -Inf | down |
| MSTRG.980155.15 | 0.00039 | -Inf | down |
| MSTRG.95360.15 | 0.043806 | -Inf | down |
| MSTRG.893754.15 | 0.047041 | -3.2831 | down |
| MSTRG.844972.3 | 0.000819 | -6.98439 | down |
| MSTRG.80030.63 | 0.03211 | -3.48642 | down |
| MSTRG.752109.47 | 5.05E-05 | -Inf | down |
| MSTRG.685856.25 | 0.001124 | -Inf | down |
| MSTRG.685518.32 | 0.01762 | -4.43069 | down |
| MSTRG.6687.20 | 0.044202 | -Inf | down |
| MSTRG.595683.34 | 0.047644 | -Inf | down |
| MSTRG.561854.49 | 0.001194 | -Inf | down |
| MSTRG.561532.38 | 0.002077 | -Inf | down |
| MSTRG.545186.4 | 0.000153 | -Inf | down |
| MSTRG.494241.37 | 0.043143 | -Inf | down |
| MSTRG.429372.8 | 0.004234 | -Inf | down |
| MSTRG.429372.19 | 0.023765 | -Inf | down |
| MSTRG.429372.18 | 0.007372 | -Inf | down |
| MSTRG.429370.3 | 0.014249 | -4.47408 | down |
| MSTRG.349808.8 | 0.014275 | -Inf | down |
| MSTRG.260129.62 | 0.04043 | -3.93548 | down |
| MSTRG.247052.131 | 0.043031 | -Inf | down |
| MSTRG.163201.87 | 0.00105 | -3.56619 | down |
| MSTRG.1373338.172 | 0.04397 | -Inf | down |
| MSTRG.1373338.124 | 0.038789 | -Inf | down |
| MSTRG.1363133.8 | 0.000182 | -Inf | down |
| MSTRG.13457.70 | 0.016755 | -Inf | down |
| MSTRG.1283690.82 | 0.046129 | -10.73 | down |
| MSTRG.1222550.19 | 0.001945 | -Inf | down |
| MSTRG.1201985.2 | 0.04959 | -Inf | down |
| MSTRG.115720.42 | 0.009539 | -3.38768 | down |
| MSTRG.1067349.42 | 0.002163 | -Inf | down |
| MSTRG.1066722.55 | 0.00793 | -2.80692 | down |
| MSTRG.1044601.12 | 0.007633 | -Inf | down |
| AP000347.2-202 | 0.002435 | -Inf | down |
| MSTRG.62509.8 | 0.046348 | -Inf | down |
| MSTRG.539573.10 | 0.038748 | -3.10612 | down |
| MSTRG.876829.7 | 0.039208 | -Inf | down |
| MSTRG.190771.21 | 0.006462 | -5.76684 | down |
| MSTRG.810520.26 | 0.001353 | -Inf | down |
| MSTRG.703759.4 | 0.023316 | -Inf | down |
| MSTRG.668360.15 | 0.001339 | -Inf | down |
| MSTRG.1372519.1 | 0.011528 | -Inf | down |
| MSTRG.1341188.2 | 0.005698 | -5.72671 | down |
| MSTRG.1276893.16 | 0.009826 | -Inf | down |
| MSTRG.1202417.42 | 0.001812 | -4.34825 | down |
| MSTRG.1196158.104 | 0.025892 | -8.09854 | down |
| MSTRG.1068754.98 | 0.039323 | -Inf | down |
| MSTRG.1068754.72 | 1E-04 | -6.11643 | down |
| MSTRG.733623.26 | 0.014636 | -3.22986 | down |
| MSTRG.177832.10 | 0.013452 | -3.11517 | down |
| MSTRG.783530.106 | 0.042426 | -Inf | down |
| MSTRG.876829.31 | 0.044717 | -Inf | down |
| MSTRG.788069.2 | 0.022481 | -2.92494 | down |
| MSTRG.432644.45 | 0.004759 | -Inf | down |
| MSTRG.1179892.56 | 0.011263 | -2.99224 | down |
| MSTRG.1319497.23 | 0.042408 | -Inf | down |
| MSTRG.979666.27 | 0.004368 | -Inf | down |
| MSTRG.891911.1 | 0.017722 | -Inf | down |
| MSTRG.880192.55 | 0.028793 | -3.74136 | down |
| MSTRG.874430.72 | 0.044329 | -Inf | down |
| MSTRG.700846.24 | 0.001879 | -8.49071 | down |
| MSTRG.507925.17 | 0.018397 | -Inf | down |
| MSTRG.24423.3 | 0.039135 | -Inf | down |
| MSTRG.235491.88 | 0.04369 | -Inf | down |
| MSTRG.175045.1 | 2.86E-06 | -Inf | down |
| MSTRG.153922.7 | 0.021017 | -3.01566 | down |
| MSTRG.1518513.56 | 0.043485 | -5.7049 | down |
| MSTRG.1336447.5 | 0.008928 | -Inf | down |
| MSTRG.1216919.21 | 0.0217 | -4.25387 | down |
| MSTRG.706722.47 | 0.000545 | -6.49529 | down |
| MSTRG.66466.76 | 0.002082 | -8.75807 | down |
| MSTRG.1184274.14 | 0.038725 | -Inf | down |
| MSTRG.354530.20 | 0.021146 | -6.13676 | down |
| MSTRG.1314749.47 | 0.002541 | -Inf | down |
| MSTRG.891911.3 | 0.040981 | -2.93817 | down |
| MSTRG.880388.22 | 0.000589 | -Inf | down |
| MSTRG.743725.37 | 0.000529 | -4.90899 | down |
| MSTRG.676775.13 | 0.028157 | -Inf | down |
| MSTRG.674800.31 | 0.001265 | -9.02594 | down |
| MSTRG.661988.85 | 0.043158 | -Inf | down |
| MSTRG.64895.80 | 5.49E-05 | -Inf | down |
| MSTRG.641478.3 | 0.046439 | -2.32717 | down |
| MSTRG.610942.62 | 0.035404 | -Inf | down |
| MSTRG.609850.62 | 0.008043 | -Inf | down |
| MSTRG.561154.51 | 0.004898 | -Inf | down |
| MSTRG.490497.6 | 0.002092 | -Inf | down |
| MSTRG.481175.25 | 0.002334 | -Inf | down |
| MSTRG.429372.17 | 0.00214 | -Inf | down |
| MSTRG.429370.42 | 0.008267 | -4.88665 | down |
| MSTRG.429370.40 | 0.008382 | -Inf | down |
| MSTRG.20799.28 | 0.034071 | -Inf | down |
| MSTRG.1341295.2 | 0.028346 | -Inf | down |
| MSTRG.1336322.32 | 0.044787 | -Inf | down |
| MSTRG.1222025.71 | 0.024322 | -Inf | down |
| MSTRG.1201972.2 | 0.045881 | -Inf | down |
| MSTRG.115589.81 | 0.034691 | -Inf | down |
| MSTRG.937216.5 | 0.001945 | -4.18259 | down |
| MSTRG.766467.123 | 0.005062 | -Inf | down |
| MSTRG.673810.17 | 0.012885 | -2.89353 | down |
| MSTRG.571069.25 | 0.028009 | -3.15923 | down |
| MSTRG.542002.161 | 0.047944 | -5.20091 | down |
| MSTRG.429370.34 | 0.00266 | -Inf | down |
| MSTRG.33837.21 | 0.018925 | -3.66854 | down |
| MSTRG.305261.15 | 0.030735 | -Inf | down |
| MSTRG.296917.28 | 0.013959 | -4.93125 | down |
| MSTRG.1283690.86 | 0.04081 | -Inf | down |
| MSTRG.1228729.48 | 2.9E-05 | -5.05627 | down |
| MSTRG.1214301.25 | 0.047347 | -2.33189 | down |
| MSTRG.1176055.2 | 0.043834 | -Inf | down |
| MSTRG.1048212.100 | 0.049073 | -Inf | down |
| MSTRG.439169.16 | 2.39E-05 | -Inf | down |
| MSTRG.634792.46 | 0.027602 | -2.96257 | down |
| MSTRG.631640.7 | 0.016411 | -2.89578 | down |
| MSTRG.627239.24 | 0.033447 | -Inf | down |
| MSTRG.450395.170 | 0.006785 | -Inf | down |
| MSTRG.339846.13 | 0.007711 | -Inf | down |
| MSTRG.667092.36 | 0.000204 | -Inf | down |
| MSTRG.161783.1 | 0.029928 | -4.18611 | down |
| MSTRG.705569.54 | 0.001121 | -Inf | down |
| MSTRG.494241.42 | 0.02982 | -Inf | down |
| NEAT1-203 | 0.003357 | -Inf | down |
| MSTRG.848607.16 | 0.001022 | -Inf | down |
| MSTRG.676950.84 | 0.0414 | -2.65718 | down |
| MSTRG.1447356.5 | 0.007868 | -Inf | down |
| MSTRG.865821.1 | 0.045881 | -Inf | down |
| MSTRG.1494844.8 | 0.032862 | -9.78488 | down |
| MSTRG.1494844.10 | 0.033902 | -Inf | down |
| MSTRG.266589.1 | 0.007887 | -6.19759 | down |
| MSTRG.705165.76 | 0.048916 | -Inf | down |
| MSTRG.661988.71 | 0.019662 | -Inf | down |
| MSTRG.1568573.9 | 0.012543 | -Inf | down |
| MSTRG.1568573.6 | 0.022689 | -Inf | down |
| MSTRG.1562716.5 | 0.011028 | -8.22212 | down |
| MSTRG.1460840.48 | 0.007389 | -5.73695 | down |
| MSTRG.1388138.12 | 0.000566 | -Inf | down |
| MSTRG.1360566.57 | 0.003493 | -8.08956 | down |
| MSTRG.650258.2 | 0.045425 | -5.53841 | down |
| MSTRG.631387.1 | 0.042667 | -Inf | down |
| MSTRG.331514.143 | 0.034437 | -Inf | down |
| HCP5-204 | 0.006761 | -Inf | down |
| MSTRG.818647.55 | 0.004995 | -Inf | down |
| MSTRG.62228.21 | 0.025761 | -Inf | down |
| MSTRG.1435009.17 | 0.000123 | -7.57424 | down |
| MSTRG.1031848.3 | 0.03838 | -Inf | down |
| MSTRG.1028640.22 | 0.00067 | -9.12047 | down |
| MSTRG.676524.26 | 9.81E-05 | -5.16257 | down |
| MSTRG.1357008.140 | 0.029856 | -2.33123 | down |
| MSTRG.1216919.24 | 0.003315 | -Inf | down |
| MSTRG.494241.44 | 0.00395 | -Inf | down |
| MSTRG.673810.97 | 0.043101 | -Inf | down |
| MSTRG.668524.12 | 0.024455 | -3.76025 | down |
| MSTRG.561410.61 | 0.040579 | -Inf | down |
| MSTRG.481547.64 | 0.026475 | -Inf | down |
| MSTRG.247052.121 | 0.04767 | -Inf | down |
| MSTRG.1474777.60 | 0.042803 | -Inf | down |
| MSTRG.1456373.57 | 0.020731 | -3.33659 | down |
| MSTRG.1263035.20 | 0.04526 | -Inf | down |
| MSTRG.673810.50 | 0.002043 | -3.52318 | down |
| MSTRG.409396.48 | 0.033193 | -9.88755 | down |
| MSTRG.200642.18 | 0.037761 | -Inf | down |
| MSTRG.484748.6 | 0.000128 | -Inf | down |
| MSTRG.1281040.29 | 0.047961 | -Inf | down |
| MSTRG.1093798.5 | 0.046262 | 5.18946 | down |
| MSTRG.1513646.4 | 0.025712 | -2.54436 | down |

Note: CeRNA, competitive endogenous RNA; lncRNA, long noncoding RNA; FC, fold change; Inf, infinity.

**Table 4.** Differentially expressed levels of 16 circRNAs in the ceRNA network.

| **CircRNAs Symbol** | ***p* value** | **log2FC** | **Regulated** |
| --- | --- | --- | --- |
| hsa_intergenic_167 | 0.0486 | Inf | up |
| hsa_CDK8_1 | 0.0251 | Inf | up |
| hsa_PFKFB4_1 | 0.0038 | Inf | up |
| hsa_NSMCE2_2 | 0.0165 | Inf | up |
| hsa_RABGAP1_20 | 0.0011 | Inf | up |
| hsa_RUFY1_1 | 0.0113 | -1.4401 | down |
| hsa_intergenic_20 | 0.0374 | -Inf | down |
| hsa_intergenic_526 | 0.0338 | -2.1911 | down |
| hsa_intergenic_570 | 0.0038 | -Inf | down |
| hsa_REV3L_16 | 0.0087 | -Inf | down |
| hsa_CD226_6 | 0.0002 | -Inf | down |
| hsa_SCFD2_6 | 0.0308 | -2.0883 | down |
| hsa_intergenic_234 | 0.0340 | -Inf | down |
| hsa_VEGFC_8 | 0.0308 | -Inf | down |
| hsa_intergenic_773 | 0.0112 | -4.0323 | down |
| hsa_HLA-B_1 | 0.0112 | -Inf | down |

Note: CeRNA, competitive endogenous RNA; circRNA, circular RNAs; FC, fold change; Inf, infinity.

**Table 5.** Differentially expressed levels of 24 miRNAs in the ceRNA network.

| **miRNA Symbol** | ***p* value** | **log2FC** | **Regulated** |
| --- | --- | --- | --- |
| novel_miR_1016 | 0.0016 | 5.7288 | up |
| novel_miR_93 | 0.0166 | 4.2394 | up |
| novel_miR_821 | 0.0039 | 2.5061 | up |
| novel_miR_76 | 0.0025 | 10.0225 | up |
| novel_miR_728 | 0.0087 | 2.1390 | up |
| novel_miR_662 | 0.0083 | 2.2618 | up |
| novel_miR_426 | 0.0098 | 4.8246 | up |
| novel_miR_363 | 0.0196 | 4.2569 | up |
| novel_miR_186 | 0.0110 | 4.4627 | up |
| hsa-miR-6850-5p | 0.0236 | 3.1132 | up |
| hsa-miR-6747-3p | 0.0171 | 1.6794 | up |
| hsa-miR-4732-3p | 0.0029 | 1.9729 | up |
| hsa-miR-4525 | 0.0268 | 4.5973 | up |
| hsa-miR-4467 | 0.0047 | 2.4003 | up |
| hsa-miR-3940-3p | 0.0148 | 1.6321 | up |
| hsa-miR-3620-3p | 0.0054 | 4.2883 | up |
| hsa-miR-216a-3p | 0.0000 | 8.8203 | up |
| hsa-miR-1303 | 0.0151 | 1.7490 | up |
| hsa-miR-1269b | 0.0040 | 9.8387 | up |
| hsa-miR-1180-3p | 0.0003 | 2.6048 | up |
| novel_miR_936 | 0.0131 | -3.0844 | down |
| novel_miR_747 | 0.0456 | -2.3132 | down |
| novel_miR_746 | 0.0456 | -2.3132 | down |
| hsa-miR-652-5p | 0.0007 | -2.0929 | down |

Note: CeRNA, competitive endogenous RNA; miRNA, MicroRNA; FC, fold change; Inf, infinity.

**Supplementary File**

**Supplemental File 1. Whole-transcriptome sequencing process.**

The extraction of total RNA from the plasma samples relied on the miRNeasy Mini Kit (Qiagen, Hilden, Germany) according to the manufacturer’s protocol. The RNA degradation and contamination, particularly the DNA contamination, was monitored on 1.5% agarose gels. The RNA concentration and purity were measured using the NanoDrop 2000 Spectrophotometer (Thermo Fisher Scientific, Wilmington, DE, USA). The RNA integrity was assessed using the RNA Nano 6000 Assay Kit of the Agilent Bioanalyzer 2100 System (Agilent Technologies, CA, USA).

For the lncRNA sequencing, a total of 1.5-μg RNA per sample was used as the input material for ribosomal RNA (rRNA) removal using the Ribo-Zero rRNA Removal Kit (Epicentre, Madison, WI, USA). Sequencing libraries were generated using NEBNext^R^ Ultra^TM^ Directional RNA Library Prep Kit for Illumina^R^ (NEB, USA) according to the manufacturer’s recommendations, and the index codes were added to attribute sequences to each sample. The clustering of the index-coded samples was performed on a cBot Cluster Generation System using TruSeq PE Cluster Kitv3-cBot-HS (Illumia) according to the manufacturer’s instructions. Following cluster generation, the library preparations were sequenced on an Illumina NovaSeq 6000 platform and reads were generated. Raw data (raw reads) in the fastq format were first processed through in-house perl scripts. During this step, clean data (clean reads) were obtained by removing from the raw data reads containing the adapter, reads containing ploy-N and low-quality reads.

Additionally, for small RNA (sRNA) sequencing, a total of 2.5-ng RNA per sample was used as the input material for the RNA sample preparation. Sequencing libraries were generated using NEBNext^R^ Ultra^TM^ small RNA Sample Library Prep Kit for Illumina^R^ (NEB, USA) adhering to the manufacturer’s recommendations, and the index codes were added to attribute sequences for each sample. The clustering of the index-coded samples and sequencing process were the same as the lncRNA sequencing. Raw data (raw reads) from the fastq format were first processed through in-house perl scripts. During this step, clean data (clean reads) were obtained by removing from the raw data reads containing the adapter, reads containing ploy-N and low-quality reads, and the reads were trimmed and cleaned by removing sequences smaller than 15 nt or longer than 35 nt. The Q20, Q30 and GC content of all clean data were calculated. Then, all downstream analyses were based on clean data with a high quality.

**Supplemental File 2. Clean data process.**

After sequencing and quality control, the analytical process for the clean data (clean reads) was carried out as followed. a) For mRNAs data, these clean reads were then mapped to the reference genome sequence. Only reads with a perfect match were further analysed and annotated based on the reference genome. The HISAT2 software (Kim et al., 2015) was used to map the data using the reference genome. The mRNAs expression levels were estimated by Fragments per Kilobase of exon model per million mapped fragments (FPKM). b) As with lncRNA identification (Han et al., 2017), the transcriptome was assembled using the StringTie (Pertea et al., 2016) based on the reads mapped to the reference genome. The assembled transcripts were annotated using the gffcompare program (Pertea et al, 2020). The known lncRNAs were differentiated from the assembled transcripts if the sequencing species had known lncRNA annotations. Unknown transcripts were used to screen for putative lncRNAs. Three computational approaches, including CPC2/CNCI/Pfam/CPAT (Sun L et al., 2013; Wang L et al., 2013; Finn RD et al., 2014; Kong L et al., 2007), were combined to sort the nonprotein-coding RNA candidates from the putative protein-coding RNAs in the unknown transcripts. Putative protein-coding RNAs were filtered out using a minimum length and exon number threshold. Transcripts with lengths of more than 200 nt and with more than two exons were selected as lncRNA candidates and further screened using CPC2/CNCI/Pfam/CPAT (Sun L et al., 2013; Wang L et al., 2013; Finn RD et al., 2014; Kong L et al., 2007), which had the power to distinguish protein-coding genes from noncoding genes. Alongside the different types of lncRNAs lincRNA, intronic lncRNA, anti-sense lncRNA and sense lncRNA were selected using cuffcompare. StringTie (1.3.1) (Pertea et al., 2016) was used to calculate FPKMs of lncRNAs. c) For circRNA identification, the circRNA identifier (CIRI) tools (Gao Y et al., 2016) and find_circ software (Memczak S et al., 2013) were used to identify circRNA. CIRI scanned the sequence alignment format (SAM) files twice and then collected sufficient information to identify and characterise circRNAs. The expression of circRNA was determined by spliced reads per billion mapping (SRPBM) through the number of junction reads identified using the CIRI tools and find_circ software. d) For the miRNA data, using the Bowtie software tools (Langmead B et al., 2009), clean reads were compared to the Silva database, the GtRNAdb database, the Rfam database and the Repbase database, respectively, to identify the sequence alignment, and then filtered for ribosomal RNA (rRNA), transfer RNA (tRNA), small nuclear RNA (snRNA), small nucleolar RNA (snoRNA) and other ncRNA and repeats (Wang XJ et al., 2021). The remaining reads were used to detect known miRNA and novel miRNA predicted through comparison with known miRNAs from the miRbase database (Kozomara A et al., 2019) and miRDeep2 tools (Friedländer MR et al., 2012). The miRNA expression levels were estimated for each sample using transcripts per million (TPM).

**REFERENCES**

Finn RD, Bateman A, Clements J, Coggill P, Eberhardt RY, Eddy SR, Heger A, Hetherington K, Holm L, Mistry J et al. (2014).Pfam: the protein families database. Nucleic acids research. 42(Database issue):D222-230. doi: 10.1093/nar/gkt1223.

Friedländer MR, Mackowiak SD, Li N, Chen W, Rajewsky N. (2012).miRDeep2 accurately identifies known and hundreds of novel microRNA genes in seven animal clades. Nucleic acids research. 40(1):37-52. doi: 10.1093/nar/gkr688.

Gao Y, Wang J, Zhao F. (2015).CIRI: an efficient and unbiased algorithm for de novo circular RNA

identification. Genome biology. 16(1):4. doi: 10.1186/s13059-014-0571-3.

Han DX, Sun XL, Fu Y, Wang CJ, Liu JB, Jiang H, Gao Y, Chen CZ, Yuan B, Zhang JB. (2017).Identification of long non-coding RNAs in the immature and mature rat anterior pituitary. Scientific reports. 7(1):17780. doi: 10.1038/s41598-017-17996-6.

Kim D, Langmead B, Salzberg SL. (2015). HISAT: a fast spliced aligner with low memory requirements. Nature methods. 12(4):357-360. doi: [10.1038/nmeth.3317](https://doi.org/10.1038/nmeth.3317).

Kong L, Zhang Y, Ye ZQ, Liu XQ, Zhao SQ, Wei L, Gao G. (2007).CPC: assess the protein-coding potential of transcripts using sequence features and support vector machine. Nucleic acids research. 35(Web Server issue):W345-349. doi: 10.1093/nar/gkm391.

Kozomara A, Birgaoanu M, Griffiths-Jones S. (2019).miRBase: from microRNA sequences to function. Nucleic acids research.47 (D1):D155-d162. doi: 10.1093/nar/gky1141.

Pertea M, Kim D, Pertea GM, Leek JT, Salzberg SL. (2016).Transcript-level expression analysis of RNA-Seq experiments with HISAT, StringTie and Ballgown. Nature protocols. 11(9):1650-1667. doi: 10.1038/nprot.2016.095.

Sun L, Luo H, Bu D, Zhao G, Yu K, Zhang C, Liu Y, Chen R, Zhao Y. (2013).Utilizing sequence intrinsic composition to classify protein-coding and long non-coding transcripts. Nucleic acids research. 41(17):e166. doi: 10.1093/nar/gkt646.

Wang L, Park HJ, Dasari S, Wang S, Kocher JP, Li W. (2013).CPAT: Coding-Potential Assessment Tool using an alignment-free logistic regression model. Nucleic acids research. 41(6):e74. doi:

10.1093/nar/gkt006.

Langmead B, Trapnell C, Pop M, Salzberg SL. (2009).Ultrafast and memory-efficient alignment of short DNA sequences to the human genome. Genome biology. 10(3):R25. doi: 10.1186/gb-2009-10-3-r25.

Wang XJ, Gao J, Wang Z, Yu Q. (2021). Identification of a Potentially Functional microRNA-mRNA Regulatory Network in Lung Adenocarcinoma Using a Bioinformatics Analysis. Frontiers in cell and developmental biology. 9:641840. doi: 10.3389/fcell.2021.641840.
